# Mechanical and transport properties of concrete incorporating recycled crushed clay bricks as coarse and fine aggregates

**DOI:** 10.1038/s41598-025-16833-5

**Published:** 2025-08-28

**Authors:** Ahmed M. N. AbdElMoaty, Hatem H. A. Ibrahim, Mohamed K. Ismail

**Affiliations:** Department of Structural Engineering, Faculty of Engineering, Cairo University, Giza, Egypt

**Keywords:** Crushed clay bricks, Recycled concrete, Mechanical properties, Impact and abrasion resistance, Non-destructive testing, Engineering, Environmental sciences, Materials science

## Abstract

Recycling crushed clay bricks as both coarse and fine aggregates has shown promising potential for producing eco-friendly concrete, helping to reduce the industry’s environmental footprint while promoting the sustainable reuse of waste materials. However, the inherent variability of these aggregates can lead to inconsistent concrete performance, emphasizing the need for a thorough investigation to assess their suitability for construction applications. For this purpose, a number of concrete mixtures incorporating crushed clay bricks as coarse and/or fine aggregates were produced and tested in this study. Specifically, four mixtures incorporating crushed clay coarse aggregate (CCCA) and another four incorporating crushed clay fine aggregate (CCFA), each at replacement levels of 25%, 50%, 75%, and 100% (by volume). Additionally, one mixture was fully developed using both CCCA and CCFA. For comparison, a control mixture containing 100% natural coarse and fine aggregates was also tested. The properties evaluated for all the developed mixtures included slump, dry density, water absorption, sorptivity, compressive strength, splitting tensile strength, flexural strength, abrasion resistance, impact resistance, ultrasonic pulse velocity, and Schmidt rebound hammer. All results were statistically analyzed to assess the effect of CCCA and/or CCFA on the test outcomes and their significance. The results indicated that replacement levels of CCCA up to 25% and CCFA up to 50% could offer a viable alternative to conventional natural aggregates, while minimizing the deterioration of concrete properties. At any same replacement level, CCFA generally outperformed CCCA, except in abrasion resistance, where CCCA mixtures exhibited better performance. As for the sorptivity, the CCFA improved the capillary structure of concrete leading to lower water ingress, while the CCCA resulted in larger capillary pores and higher sorptivity values compared to the control mix. Under impact loading, replacing more than 25% of the aggregates with either CCCA or CCFA resulted in a significant reduction in the energy absorption capacity of the specimens, thus limiting their suitability for applications exposed to high impact loads. However, combining both CCCA and CCFA at full replacement levels can effectively produce sustainable semi-lightweight concrete with strengths above 25 MPa, making it suitable for various structural applications, although its suitability for environments requiring high abrasion and impact resistance is limited. The findings also suggest that non-destructive tests such as the Schmidt hammer and UPV tests can be used for assessment, with the Schmidt hammer test providing more reliable results for evaluations and estimations. Statistical analysis showed that CCCA, CCFA, and their interaction significantly affected most concrete properties. However, their use resulted in higher variability than natural aggregates, especially in splitting tensile strength, abrasion, and impact energy tests. While CCCA reduces embodied energy compared to natural coarse aggregates, the use of CCFA increases it, though CCFA remains a sustainable alternative for natural sand, aiding in resource conservation.

## Introduction

 In recent years, there has been growing interest in integrating recycling strategies within the construction industry. This practice not only reduces the environmental impact of construction but also find innovative ways to reuse waste materials, thereby conserving natural resources. One of the most explored areas of research is the use of waste materials as partial or total replacements for natural aggregates in concrete. Several studies^1–15^ have investigated the potential of various waste materials—such as crushed old concrete, ceramic, glass, rubber, roof tiles, crushed clay bricks, palm oil clinker, and sewage sludge—as replacements for either coarse aggregates and/or fine aggregates. The findings from these studies indicated that while incorporating these materials into concrete production held significant potential for environmental sustainability, it often came with trade-offs in the strength and durability of the resulting concrete mixture. Therefore, having a thorough understanding of the effects of these materials is crucial in order to identify their most suitable applications.

One of the emerging waste materials obtained from demolition is waste clay bricks. These bricks, which are often discarded during the demolition of old buildings. Some studies reported that by crushing and processing these bricks, they can be used as aggregates in concrete, providing a sustainable alternative to natural aggregates. Poon and Chan^16^produced paving blocks using coarse aggregates derived from recycled crushed clay bricks. They found that the compressive strengthof the paving blocks was significantly reduced, almost half of the strength of a control mixture made with natural aggregates. Zheng et al^17^. also conducted a study to evaluate the effect of replacing coarse aggregates in concrete with crushed clay coarse aggregates (CCCA) at varying replacement levels of 0%, 25%, 50%, 75%, and 100%. This research focused on investigating the influence of CCCA on the compressive strength and microstructure of concrete. The results showed that the compressive strength of concrete decreased by 3.3%, 8.0%, 11%, and 13% at the replacement of 25%, 50%, 75%, and 100%, respectively. Similar results were observed in other studies^18,19^, where the inclusion of 100% CCCA replacement led to a reduction in splitting tensile and flexural strengths. On the other side, Adamson et al.^20^ focused on the durability effects of using CCCA as a partial replacement for coarse aggerates at replacement level of 25% and 50%. The researchers noticed that as the content of CCCA increased, the concrete’s resistance to chloride penetration decreased, while the freeze/thaw resistance improved. Ahmad and Hossain^21^ investigated the influence of CCCA on the absorption and permeability of concrete. Their findings proved that using CCCA led to an increase in both absorption and porosity of the concrete, potentially lowering its durability. Yang et al.^22^ evaluated the use of both recycled concrete aggregates and CCCA in concrete. The CCCA and recycled concrete aggregates were mixed in the following ratios: 20%:80%, 50%:50%, and 0%:100%. When compared to the control mixture made with natural coarse aggregate (NCA), the lowest compressive, splitting tensile, and flexural strengths were found in the 50%:50% combination. This mixture showed a 19.8% reduction in compressive strength, a 33.9% decrease in splitting tensile strength, and a 5.9% reduction in flexural strength. Zhang et al.^23^ also reported a decrease of 18.3% in compressive strength and a decrease of 53.1% in splitting tensile strength when 30% CCCA replacement level was used. They also reported that the presence of microcracks and pores in the cement matrix and at the aggregate interface using SEM. Hakim et al.^24^ studied concrete with varying levels of CCCA replacement (0%, 25%, 50%, 75%, and 100%) and observed a reduction in compressive strength ranging from 16 to 63%, as well as a decrease in flexural strength between 17% and 40%. With the same replacement levels, further investigation was performed by Atyia et al.^5^ to assess slump, density, permeability, sorptivity, and compressive strength. The results showed a decline in all measured properties as the CCCA content increased, yielding a reduction in the compressive strength reached up to 33% when 100% CCCA was used.

Other studies investigated the use of crushed clay bricks as a fine aggregate and powder in mortar and concrete. In mortar, Sathiparan^25^ investigated the use of clay bricks and other masonry debris as partial sand replacements at replacement levels of 25%, 50%, and 75%. As a result, they found improved compressive strength and resistance to alkaline and acidic environments; however, reductions were observed in flexural strength, impact resistance, abrasion resistance, and sorptivity. Likewise, Ibrahim et al.^26^ reported that using clay brick waste powder as a partial lime replacement in restoration mortars reduced porosity, increased resistance to salt attack, and improved drying shrinkage control. Xu et al.^27^ also showed that replacing up to 50% of natural sand with crushed clay brick fine aggregate (CCFA) improved water retention, tensile bond strength, and compressive strength—particularly at 30% replacement—though higher contents led to increased shrinkage. In another study, Mohammed et al.^28^ stated that the use of CCFA as a replacement for sand in mortar helped to reach a denser microstructure, showing the optimal replacement level was 4%, beyond which compressive strength gradually decreased. Additionally, an 8% CCFA replacement showed the highest resistance to carbonation. Additionally, O’Farrell et al.^29^ found that replacing up to 30% of natural sand with CCFA refined the pore structure but caused a near-linear reduction in compressive strength. Other studies^30,31^ further demonstrated that adding clay brick waste powder into mortars enhanced long-term strength, reduced shrinkage, and minimized alkali-silica reactivity. In concrete, Poon et al.^32^ explored the effect of replacing fine aggregate with CCFA on the compressive strength of concrete, using a replacement level of 20%. They found that this replacement caused a reduction in the compressive strength reached 18%. Fadya et al.^33^ also reported a reduction in the compressive and splitting tensile strength reached 49% and 36%, respectively, when 100% CCFA was used. Additionally, the density of the concrete decreased by up to 10% under the same replacement level. Dang et al.^34^ studied the influence of CCFA on the durability and microstructure of concrete, with replacement levels of 50% and 100%. The results revealed that increasing the replacement led to a gradual improvement in chloride resistance, unlike the effect of CCCA reported by^26^. However, they also observed a decrease in water absorption, sorptivity, drying shrinkage, and carbonation resistance, suggesting that while some durability aspects were improved, other key properties were negatively affected. Some other researchers like Aliabdo et al.^35^ investigated both CCCA and CCFA for comparison. Their research program incorporated both aggregates at replacements of 0%, 50%, 75% and 100%. They found that the use of CCCA had a consistent negative effect on strengths of concrete, while using 25% CCFA caused an improvement in compressive and splitting strengths by 9.9% and 12.2%, respectively, but further increase in CCFA had a negative impact. Ahmed^36^ recently utilized both CCCA and CCFA in the development of pervious concrete, highlighting their beneficial effects on improving permeability and reducing clogging behavior, while maintaining acceptable strength for multiple applications.

Although the aforementioned studies have made significant efforts to evaluate the potential of CCCA and CCFA in concrete, there is no clear consensus on the optimal replacement percentage. Additionally, none of the studies have comprehensively focused on evaluating important properties such as abrasion and impact resistance, which are crucial for understanding the long-term performance and durability of concrete in related applications. Furthermore, the use of non-destructive testing methods, such as ultrasonic pulse velocity (UPV) and Schmidt hammer, which can provide valuable insights into the quality and integrity of concrete without causing damage, has been largely overlooked. Recent research, however, has highlighted the strong potential of these non-destructive tests to predict key concrete properties^37–43^. The present study aimed to address this gap of knowledge by investigating a number of concrete mixtures incorporating CCCA and CCFA at different replacement levels, specifically 25%, 50%, 75%, and 100%. A comprehensive testing program was implemented to evaluate the effects of these aggregates on several key properties of concrete. These included slump, dry density, water absorption, sorptivity, compressive strength, splitting tensile strength, flexural strength, abrasion resistance, impact resistance, UPV, and Schmidt rebound hammer.

## Research significance

This research contributes to evaluating the feasibility of recycling waste clay bricks in concrete through a comprehensive assessment of incorporating CCCA and CCFA at various replacement levels. Building on existing studies, it specifically investigates their effects on concrete performance, with a focus on transport properties, abrasion resistance, and impact resistance - areas that are underexplored in the current literature. The findings offer insights into the optimal use of CCCA and CCFA, helping engineers make informed decisions for structural and nonstructural applications. Furthermore, this study advances the development of sustainable concrete by offering a viable strategy to reduce environmental waste, decrease reliance on natural aggregates, and promote circular economy principles in the construction industry.

## Methodology

**Fig. 1:**
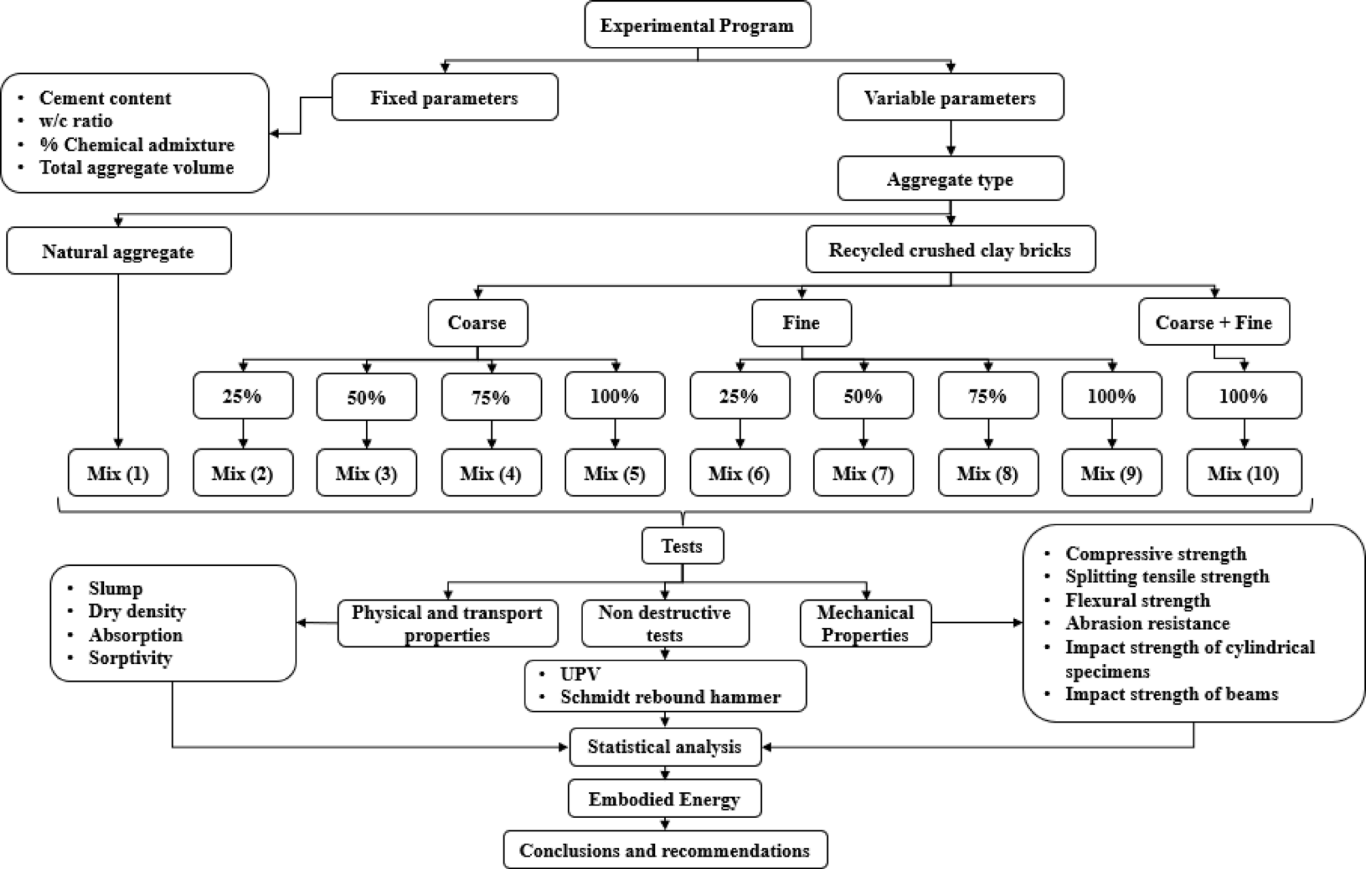
Methodology and experimental program flowchart.

To evaluate the feasibility of recycling crushed clay bricks as a replacement for natural aggregates in concrete, the following methodology was implemented, as outlined in the flow chart shown in Fig. 1. The recycled clay bricks were first cleaned, and any residual mortar was removed before being crushed into two aggregate types: CCCA and CCFA. CCCA was used to replace natural coarse aggregates, while CCFA replaced natural fine aggregates at substitution levels of 25%, 50%, 75%, and 100%. Additionally, a concrete mix combining both CCCA and CCFA was prepared. In total, nine concrete mixtures were produced and compared against a control mix containing only natural aggregates. Standardized procedures were followed for mixing, casting, curing, and testing. Fresh concrete workability was assessed using slump tests. Physical and transport properties, including dry density, water absorption, and sorptivity, were measured. Mechanical performance was evaluated through compressive, splitting tensile, and flexural strength tests. Durability was assessed using abrasion resistance and impact resistance tests. Non-destructive testing methods, such as ultrasonic pulse velocity (UPV) and Schmidt rebound hammer tests, were employed to evaluate the quality and internal integrity of the concrete. These non-destructive test results were also correlated with the mechanical property measurements. All tests adhered to relevant standards, and results were averaged over three replicates to ensure accuracy. Statistical analyses included the calculation of the coefficient of variation (*CV*) to assess data variability and one-way analysis of variance (ANOVA) to determine the significance of the effects of CCCA and CCFA replacement levels on the measured properties. Additionally, the impact of incorporating CCCA and/or CCFA on the embodied energy of the concrete mixtures was examined.

## Experimental program

### Materials

**Fig. 2:**
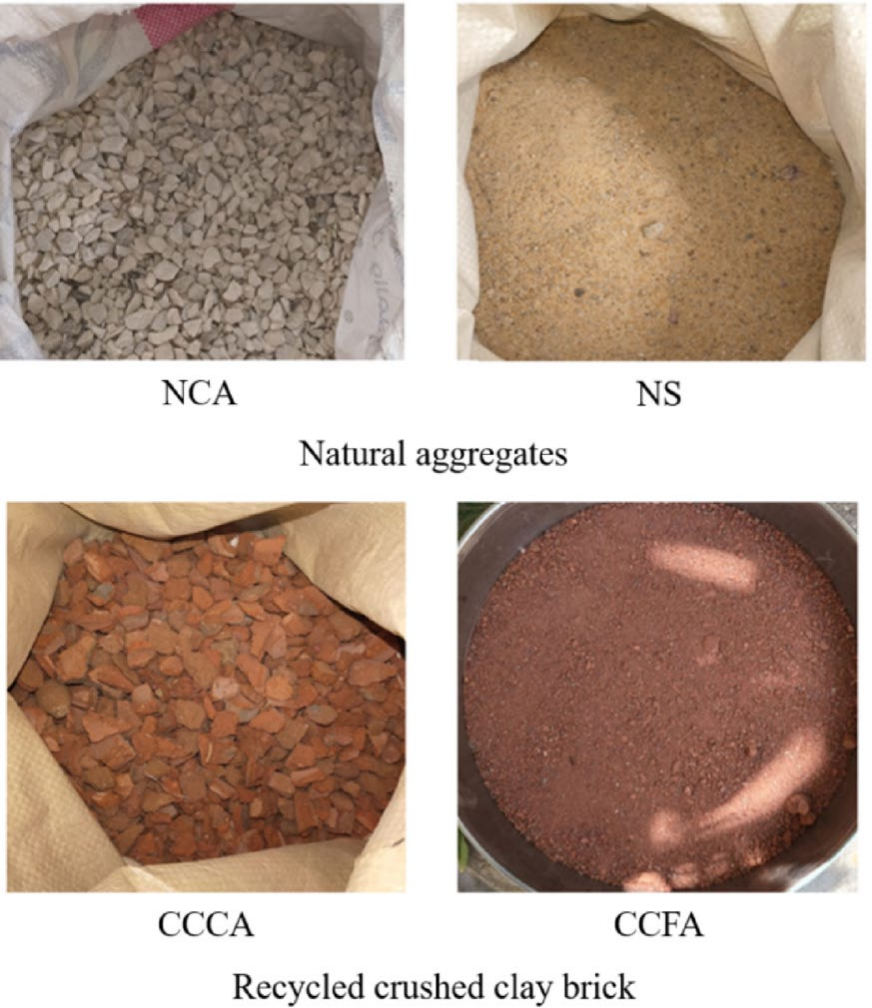
The natural and recycled aggregates used.

**Fig. 3:**
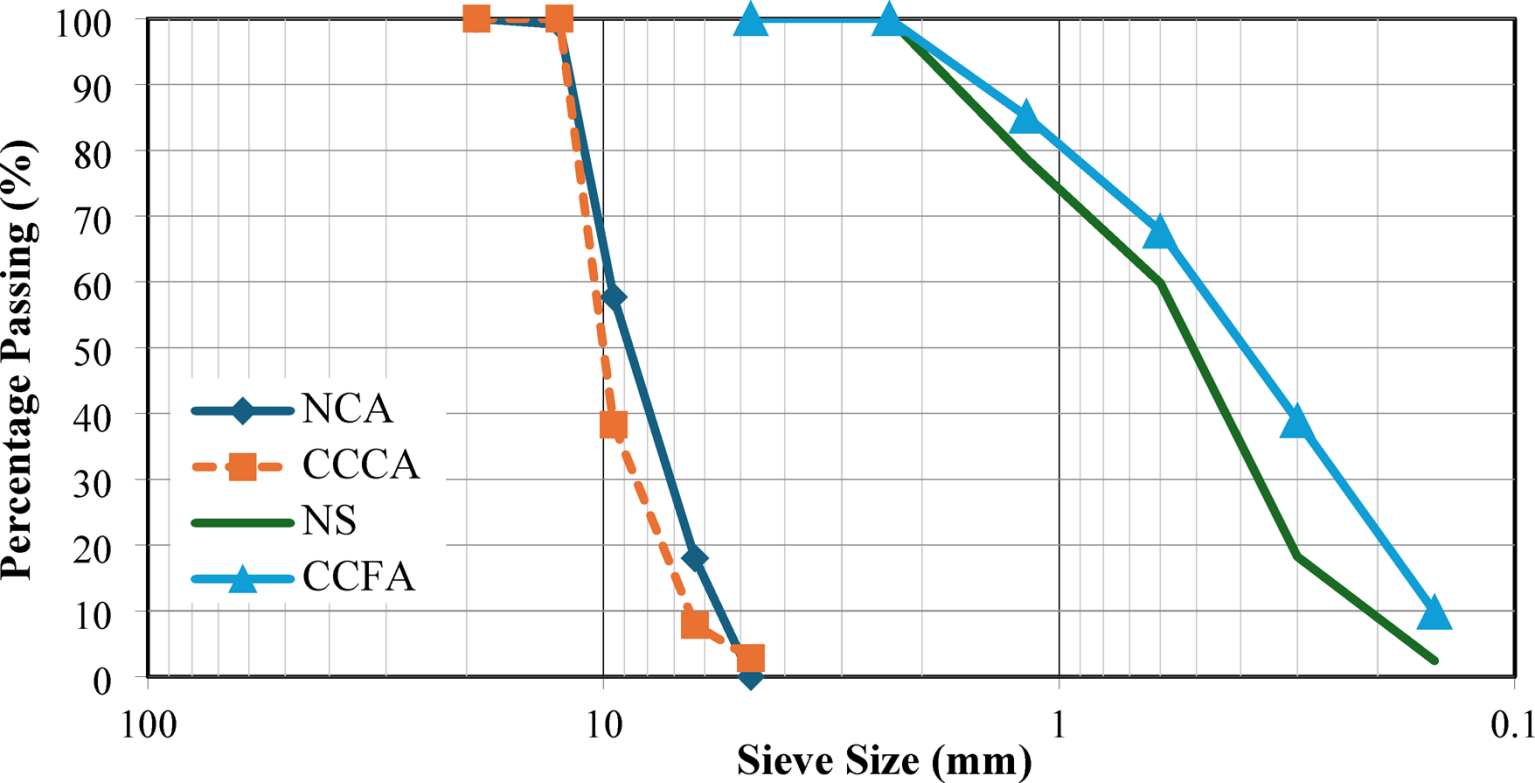
The gradation of aggregates used.

Ordinary Portland cement type CEM I-52.5 N, conforming to ASTM C150 Type 1^44^, was used as the primary binder material. Two types of aggregates were utilized (see Fig. 2): (a) natural aggregates, consisting of crushed dolomite as the NCA and natural sand (NS) as the fine aggregate; (b) recycled crushed clay bricks, which were used in both fine and coarse forms. The physical and mechanical properties of all aggregate types are summarized in Table 1, with their gradation shown in Fig. 3. A water-reducing agent, conforming to ASTM C 494^45^, was added to adjust the workability of the mixtures.

**Table 1:** Physical and mechanical properties of natural and recycled aggregates.

Properties	Specific gravity (kg/m^3^)	Fineness modulus	Maximum aggregate size (mm)	Crushing index
NCA	2.25	-	12.5	24.39
NS	2.62	2.41	-	-
CCCA	2.22	-	12.5	64.76
CCFA	2.38	2.53	-	-

### Concrete mixtures and mixing

In this study, ten concrete mixtures were developed to investigate the optimal use of CCCA and/or CCFA in concrete, as detailed below:

- One mixture, designated as M1, was produced using both natural coarse and fine aggregates (i.e., NCA and NS). This mixture served as the control for the other mixes that incorporated varying replacement levels of CCCA and/or CCFA.
- Four mixtures, designated as M2, M3, M4, and M5, respectively, were produced with different replacement levels of CCCA, specifically 25%, 50%, 75%, and 100%. These mixtures were designed to evaluate the effect of using CCCA as an alternative to natural coarse aggregates.
- Four mixtures, designated as M6, M7, M8, and M9, respectively, were developed with different replacement levels of CCFA, specifically 25%, 50%, 75%, and 100%. These mixtures aimed to assess the effect of using CCFA as an alternative to natural fine aggregates.
- One mixture, designated as M10, was entirely produced with both CCCA and CCFA to evaluate their combined effect on concrete properties.

In all mixtures, the binder content and water-to-cement ratio were kept constant at 450 kg/m³ and 0.4, respectively. The water-reducing agent was also incorporated at 1.5% of the cement content. The detailed compositions of all developed mixtures are presented in Table 2.

For the mixing process, the cement, fine aggregates, and coarse aggregates were mixed dry for 2 min. Two-thirds of the water was then added, and mixing continued for another 2 min. The remaining water, combined with the water-reducing agent, was added next, followed by 3–5 min of mixing until the desired workability was achieved. After measuring workability, the mixture was poured into molds. Specimens were demolded after 24 h and moist cured until testing.

**Table 2:** Composition of all developed concrete mixtures.

Mix #	Mixture	Cement	NCA	NS	CCCA	CCFA	Water
M1	Control	450	853.2	887.1	--	--	180
M2	25% CCCA	450	639.9	887.1	187.9	--	180
M3	50% CCCA	450	426.6	887.1	375.8	--	180
M4	75% CCCA	450	213.3	887.1	563.7	--	180
M5	100% CCCA	450	0.0	887.1	751.6	--	180
M6	25% CCFA	450	853.2	665.3	--	201.5	180
M7	50% CCFA	450	853.2	443.5	--	402.9	180
M8	75% CCFA	450	853.2	221.8	--	604.4	180
M9	100% CCFA	450	853.2	0.0	--	805.8	180
M10	100% CCCA + 100% CCFA	450	0.0	0.0	751.6	805.8	180

Note: All mixtures have a 0.4 w/b ratio, NCA = Normal Coarse Aggregate, NS = Normal Sand, CCCA = Crushed Clay Coarse Aggregate, CCFA = Crushed Clay Fine Aggregate, all numbers are in (kg/m^3^).

### Tests

All tests performed in this study are summarized in Table 3 and described in detail below.

#### Workability

The workability of all mixtures was assessed using slump test according to ASTM C143^46^.

#### Unit weight

The dry densities of all mixtures were measured as per BS EN 12390-7^47^ to investigate the effect of CCCA and CCFA on the unit weight of the concrete.

#### Water absorption and sorptivity

Three 100 mm x 100 mm x 100 mm cubes for each mixture were used to measure the water absorption. The specimens were submerged in water for 48 h, then completely dried in an oven, and weighed before and after each stage, following ASTM C642^48^.

**Fig. 4:**
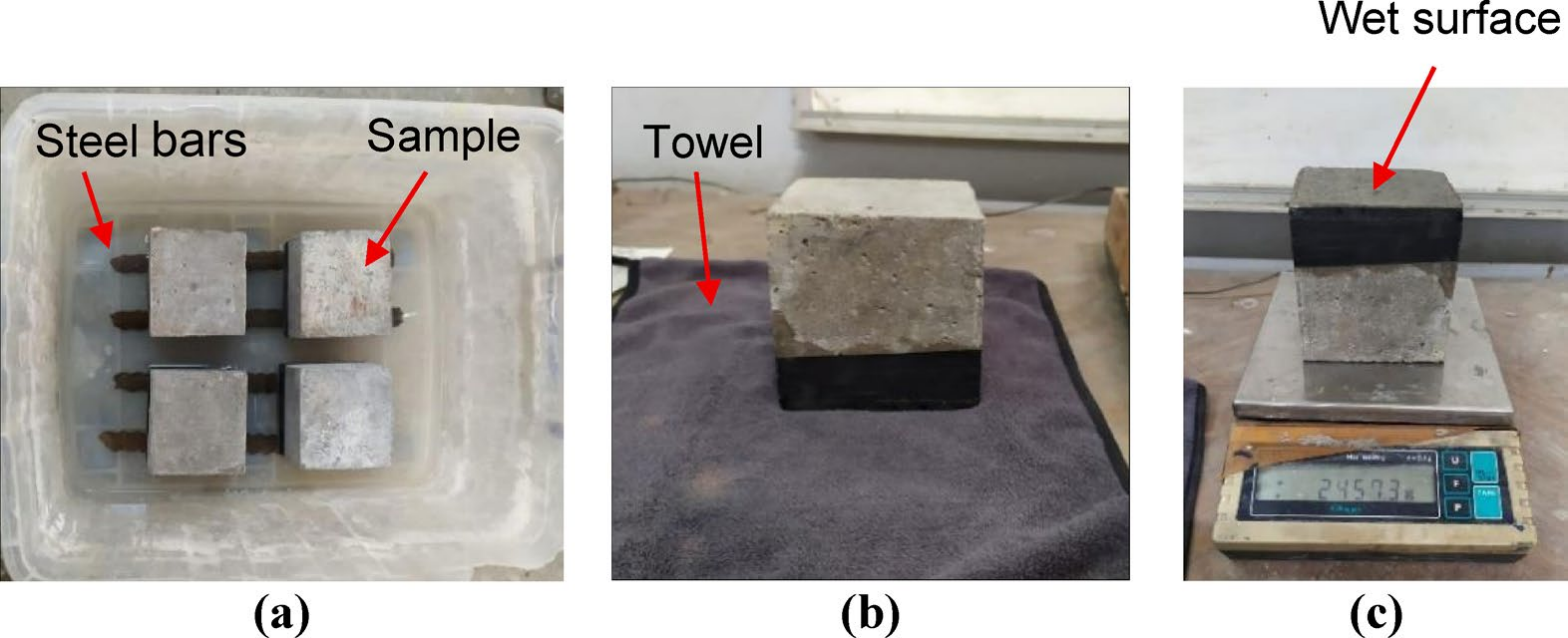
Sorptivity test: (**a**) specimens placed in container, (**b**) removing excess water from wet surface, (**c**) measuring weight change.

Furthermore, the sorptivity, according to the procedure provided in ASTM C1585^49^, was measured for each mixture. After curing for 28 days, the specimens were oven-dried at 105 °C until they reached a constant mass. After that the sides of the specimens were carefully sealed using vinyl electrician’s tape to limit the suction of water to the bottom face. Then, as shown in Fig. 4a, the specimens were placed in container over two steel bars, in which the water level covered only the lower portion (about 5 mm to 10 mm) of the specimen. At regular time intervals for weight measurement, the specimens were removed from the container, and any surface water was gently wiped off with a moist paper towel (see Fig. 4b). After wiping, the wet surface was inverted to prevent it from coming into contact with the balance pan (see Fig. 4c). Weight changes were then used to calculate the initial and secondary sorptivity values. The initial sorptivity reflects the rate of water absorption by capillary suction during the first 6 h of the test, while the secondary sorptivity measures the water absorption rate between 1 and 7 days, where the absorption slows as it becomes diffusion-controlled due to pore saturation.

#### Compressive strength, splitting tensile strength, and flexural strength tests

A compressive strength test was performed on three cubes of each mix, with dimensions 150 mm x 150 mm x 150 mm, conforming to BS EN 12,390^50^. The splitting tensile strength was evaluated using three identical cylinders of 100-mm diameter and 200-mm height as per ASTM C496^51^, while three concrete prisms of 100 × 100 × 400 mm for each mixture were used to assess the flexural strength according to ASTM C78^52^.

#### Abrasion test

**Fig. 5:**
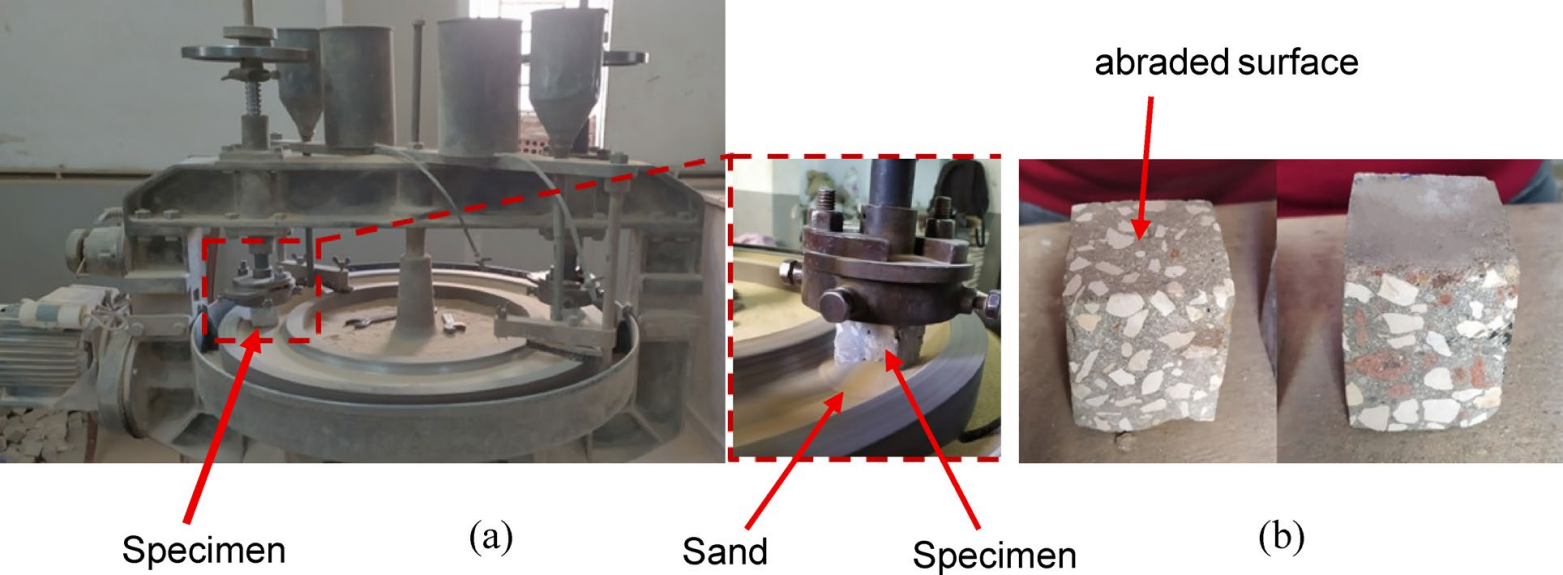
Abrasion test (**a**) test setup, (**b**) specimen before and after test.

The abrasion test was performed using the Amsler abrasive wear testing machine, following the guidelines of ABNT NBR 12,042^53^. This test was designed to simulate the effects of moving traffic on concrete surfaces and assess their ability to endure friction and mechanical wear over time. Three identical specimens, each measuring 50 mm x 50 mm x 70 mm, were tested for each mixture. As shown in Fig. 5, each specimen was placed under a weight of 66 N and positioned on a rotating abrasive disc. The disc was covered with silica sand ranging in size from 0.3 to 0.6 mm (i.e., abrasive material). The machine rotates the abrasive disc against the specimen, subjecting it to the wear process for a specific number of revolutions corresponding to a 500-meter path. It is important to note that fresh sand was used for each rotation to maintain the abrasive medium. The specimen’s weight was measured both before and after the test, and the abraded thickness was calculated accordingly. A lower thickness loss indicates better abrasion resistance, which is essential for concrete used in high-traffic areas such as pavements, roads, and floors subjected to heavy foot or vehicle traffic.

#### Impact test

**Fig. 6:**
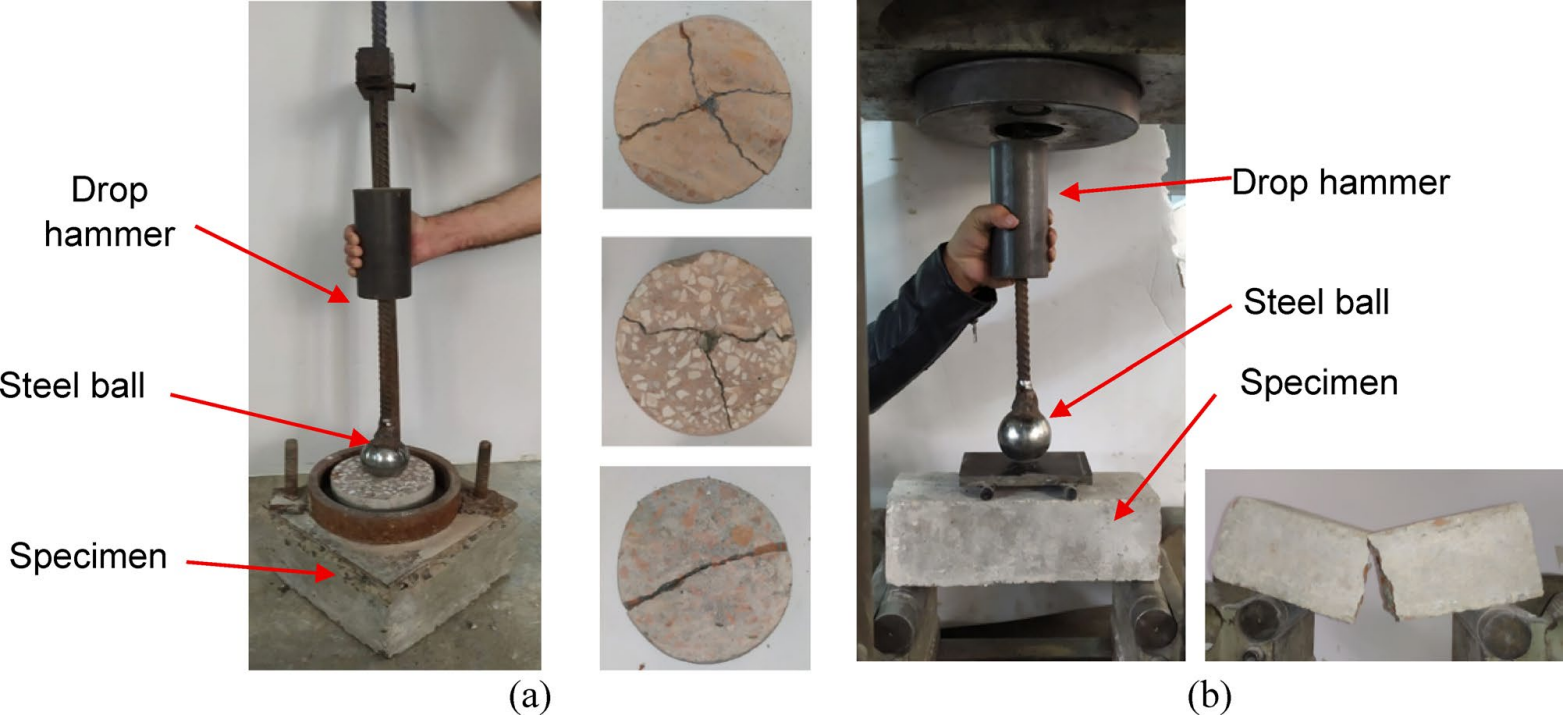
Impact tests and typical failure modes (**a**) cylindrical specimens, (**b**) beam specimens.

The impact resistance of all developed mixtures was evaluated by two different methods:

1. The method was performed as per the ACI committee 544^54^, as shown in Fig. 6a. For each mixture, three identical cylindrical specimens with a diameter of 150 mm and a thickness of 63.5 mm were tested. During the test, a 4.45 kg weight was repeatedly dropped from a height of 200 mm onto a 63.5 mm steel ball, which was positioned at the center of the specimen’s top surface. It should be noted that a height of 200 mm (i.e., smaller than the height recommended by^54^ for normal concrete) was used in this test, as the concrete becomes more brittle and exhibits lower energy absorption with the introduction of CCCA and/or CCFA. Using a larger height led to unreliable results with high deviation. Therefore, after trials using the 200 mm height, it was possible to obtain more representative results to capture the effect of CCCA and/or CCFA.
2. The second method was conducted using four-point loading setup (see Fig. 6b) to assess the energy absorption capacity under flexural impact loading. For each mixture, three identical beams with a cross-section of 100 mm x 100 mm and a loading span of 300 mm were tested. In this test, a 4.45 kg weight was repeatedly dropped from a height of 150 mm onto a steel ball placed on a steel plate, which was used to distribute the load across two loading points.

Since the failure in all mixtures was brittle and occurred suddenly, it was difficult to detect the first crack. As a result, in both tests, the impact energy that caused the failure was calculated solely using Eq. 1.

*IE = N mgh*                                                                                                                                                    (1)

#### Ultrasonic pulse velocity (UPV) test

**Fig. 7:**
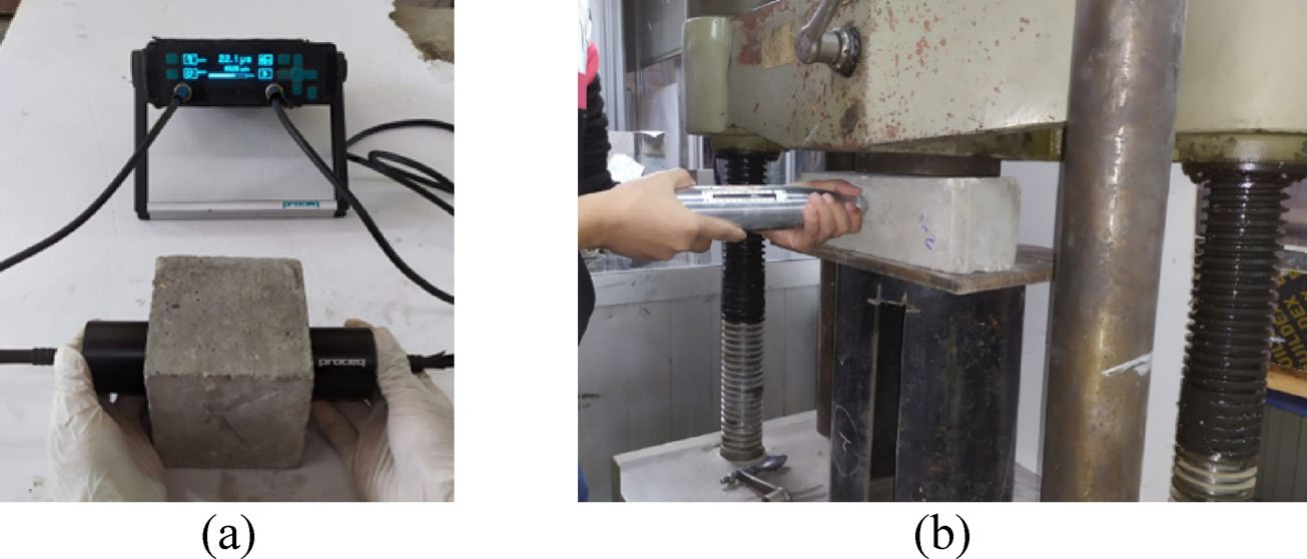
Non-destructive tests (**a**) UPV test, (**b**) Schmidt hammer test.

The UPV test was conducted in accordance with ASTM C597^55^ as a non-destructive method to evaluate the homogeneity of the mixture, as well as detect voids, defects, and cracks. Two cubes, each with dimensions of 100 mm x 100 mm x 100 mm, were used for each mix to measure UPV using the direct transmission technique, as illustrated in Fig. 7a. In this method, two ultrasonic transducers (a transmitter and receiver) were placed on opposite sides of the concrete specimen (typically represents concrete columns, beams, or walls). The transmitter emits an ultrasonic pulse through the concrete, while the receiver detects the pulse once it passes through the specimen. The pulse velocity (*V*) is determined using Eq. 2.

*V* =* L*/*T*                                                                                                                                                                (2)

where *L* is the distance between the transmitter and receiver, *T* is the time it takes for the pulse to travel the distance.

A higher pulse velocity typically indicates high-quality concrete with low porosity and fewer cracks or voids, while a lower pulse velocity may refer to poor-quality concrete with high porosity or significant defects.

#### Schmidt hammer test

The Schmidt hammer test is a non-destructive method commonly used to assess the surface hardness of concrete, which is often correlated with the concrete’s compressive strength. This test was conducted in accordance with ASTM C805^56^ on two specimens for each mixture. As shown in Fig. 7b, a concrete prism with dimensions of 100 × 100 × 400 mm was placed in a compression machine for fixation. Twelve readings were then taken on the side face of the specimen, and the average value was calculated. A higher average corresponds to a harder and stronger surface.

**Table 3:** Details of conducted tests.

Test	Specimens		Test standard
	Configuration	Dimensions	
Slump	Cone	100 mm diam. top x 200 mm diam. bottom x 300 mm height	ASTM C143 [47]
Unit weight	Cubes	100 × 100 × 100 mm	BS EN 12390-7 [48]
Water absorption	Cubes	100 × 100 × 100 mm	ASTM C642 [49]
Sorptivity	Cubes	100 × 100 × 100 mm	ASTM C1585 [50]
Compressive strength	Cubes	150 × 150 × 150 mm	BS EN 12,390 [51]
Splitting tensile strength	Cylinders	100 mm diameter, 200 mm height	ASTM C496 [52]
Flexural strength	Prisms	100 × 100 × 400 mm	ASTM C78 [53]
Abrasion resistance	Prisms	50 × 50 × 70 mm	ABNT NBR 12,042 [54]
Impact of cylindrical/discs	Discs	150 mm diameter, 63.5 mm thickness	ACI committee 544 [55]
Impact of prisms	Prisms	100 × 100 × 400 mm	ACI committee 544 [55]
UPV	Cubes	100 × 100 × 100 mm	ASTM C597 [56]
Schmidt hammer	Prisms	100 × 100 × 400 mm	ASTM C805 [57]

## Results and discussion

### Slump test

**Fig. 8:**
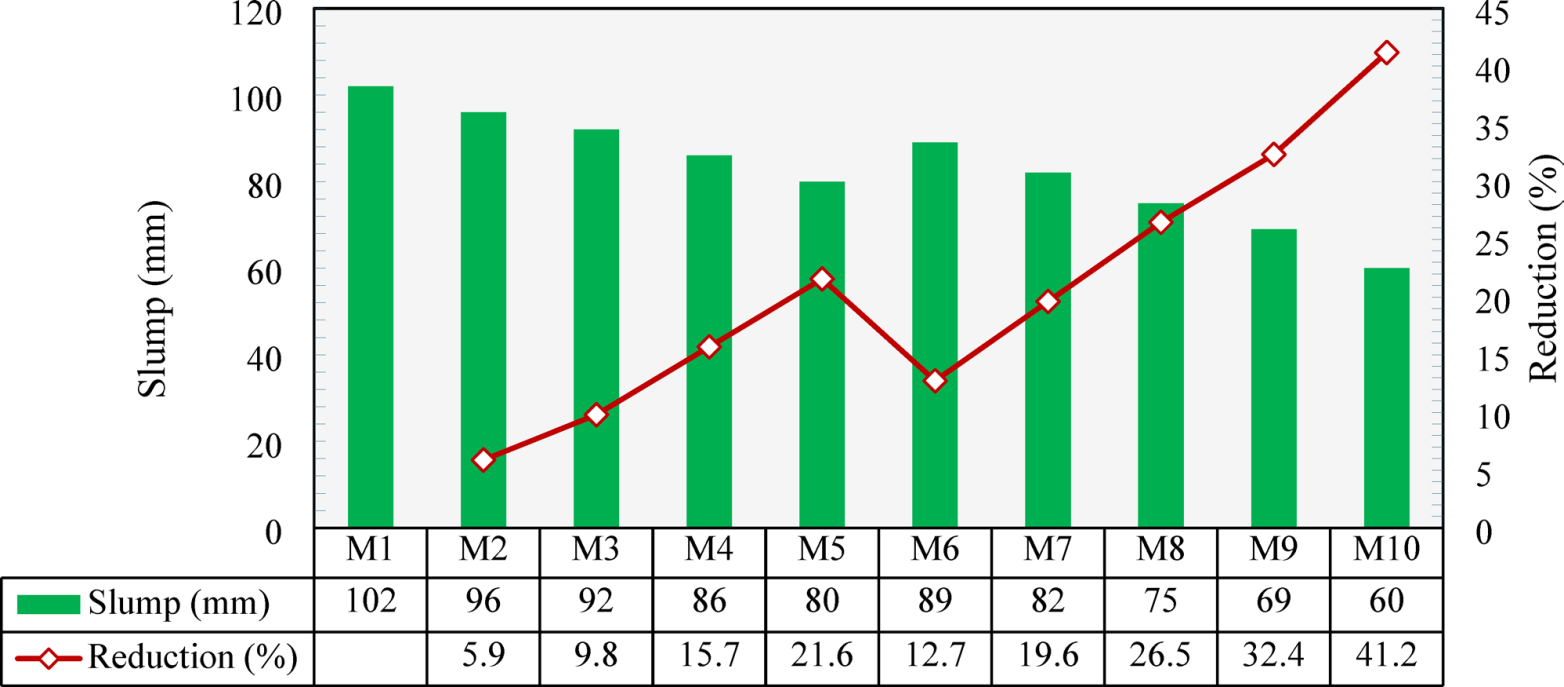
Slump test results and percentage reduction compared to the control mixture (M1).

Figure 8 presents the slump values for all the developed mixtures. The control mix (M1), made with NCA and NS, exhibited a slump value of 102 mm. When NCA was replaced with CCCA, workability started to decrease, with reductions of 5.9% and 9.8% at 25% and 50% replacement levels, respectively, as seen by comparing M2 and M3 with M1. At 75% and 100% replacement levels in M4 and M5, the reductions were 15.7% and 21.6%, respectively. These decreases could be attributed to the porous surface of CCCA compared to NCA, which consumed more mortar to fill the surface pores, thereby reducing workability. Additionally, the higher angularity of CCCA compared to NCA increased interparticle friction, further decreasing workability. Another explanation, as suggested by^28^, links the reduction in workability to the relatively weak and porous structure of CCCA, which may have caused some particles to break during mixing, thereby increasing the surface area and requiring more mortar to cover new surfaces.

Figure 8 also shows the variation in slump values for mixtures where NS was replaced by CCFA. The 25% CCFA replacement level (M6) resulted in a 12.7% reduction compared to the control mix (M1). Larger reductions were noted at higher replacement levels, with slump decreases of 19.6%, 26.5%, and 32.4% for 50%, 75%, and 100% replacements (M7, M8, and M9), respectively. This could be attributed to the higher surface area of CCFA compared to NS, as indicated by their gradations shown in Fig. 3. Mixture M10, which was developed entirely with CCCA and CCFA, exhibited the lowest slump value of 60 mm, corresponding to a 41.2% reduction compared to the control mix (M1).

**Fig. 9:**
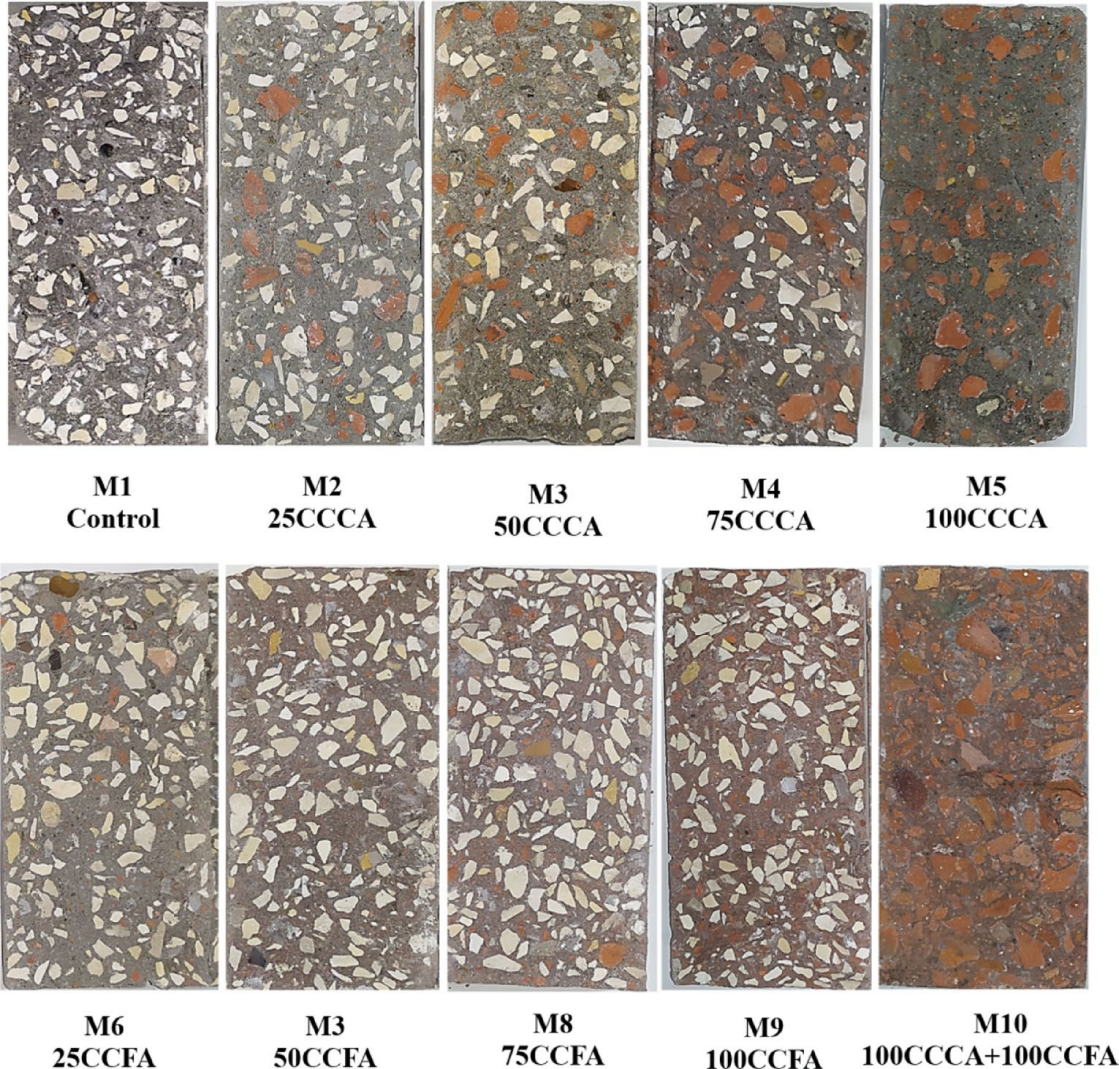
Visual inspection for aggregates distribution.

Figure 9 shows the fracture surface of concrete cylinders after a splitting test, which was used to assess the distribution of CCCA and/or CCFA in the hardened mixture. Visual inspection revealed that both CCCA and/or CCFA had a good distribution along the fracture surface, suggesting the aggregates were well mixed.

### Dry density

**Fig. 10:**
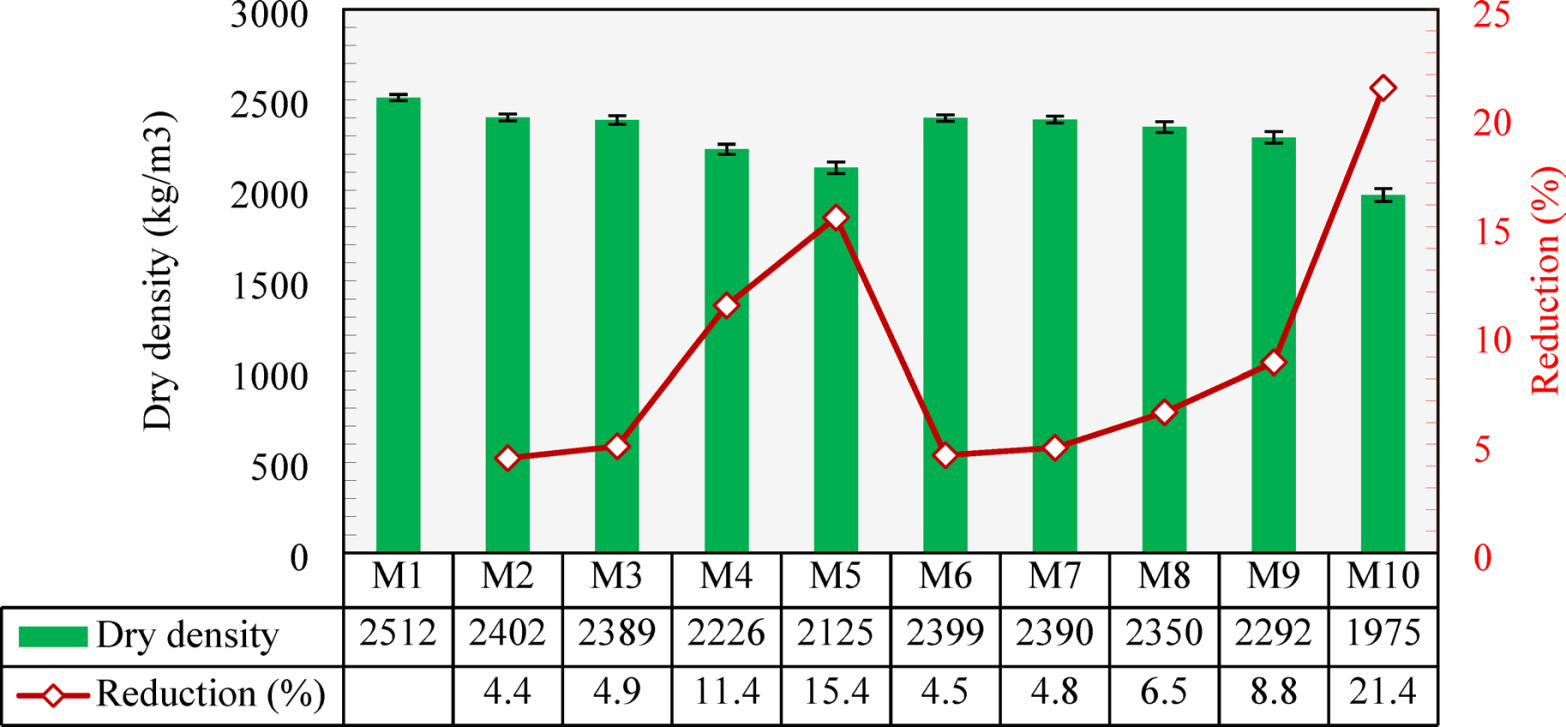
Dry density of all developed mixtures and percentage reduction compared to the control mixture (M1).

The measured dry densities of all developed mixtures are presented in Fig. 10. The results indicated that the dry density decreased as the content of either CCCA or CCFA increased. The control mix (M1) had a dry density of 2512 kg/m³. As the NCA was replaced by CCCA at 25%, 50%, 75%, and 100% (M2-M5), the dry density dropped by 4.4%, 4.9%, 11.4%, and 15.4%, respectively, compared to M1. For CCFA replacements, the 25% and 50% replacement levels showed comparable densities to those of CCCA replacements. However, at 75% and 100% replacements, CCFA resulted in a lower reduction in density compared to CCCA. This is likely due to the higher porosity of CCCA, where the water inside the pores would further dry out or be consumed in the hydration process, leading to a greater reduction in density. Combining both CCCA and CCFA in M10 resulted in a 21.4% reduction compared to M1. According to CSA^52^, concrete with a dry density ranging from 1850 to 2150 kg/m³ is categorized as semi-lightweight concrete. As a result, mixtures M5 and M10 fall within this range, demonstrating promising potential for structural applications that require reduced self-weight, ultimately leading to cost savings and more economic designs.

### Water absorption

**Fig. 11:**
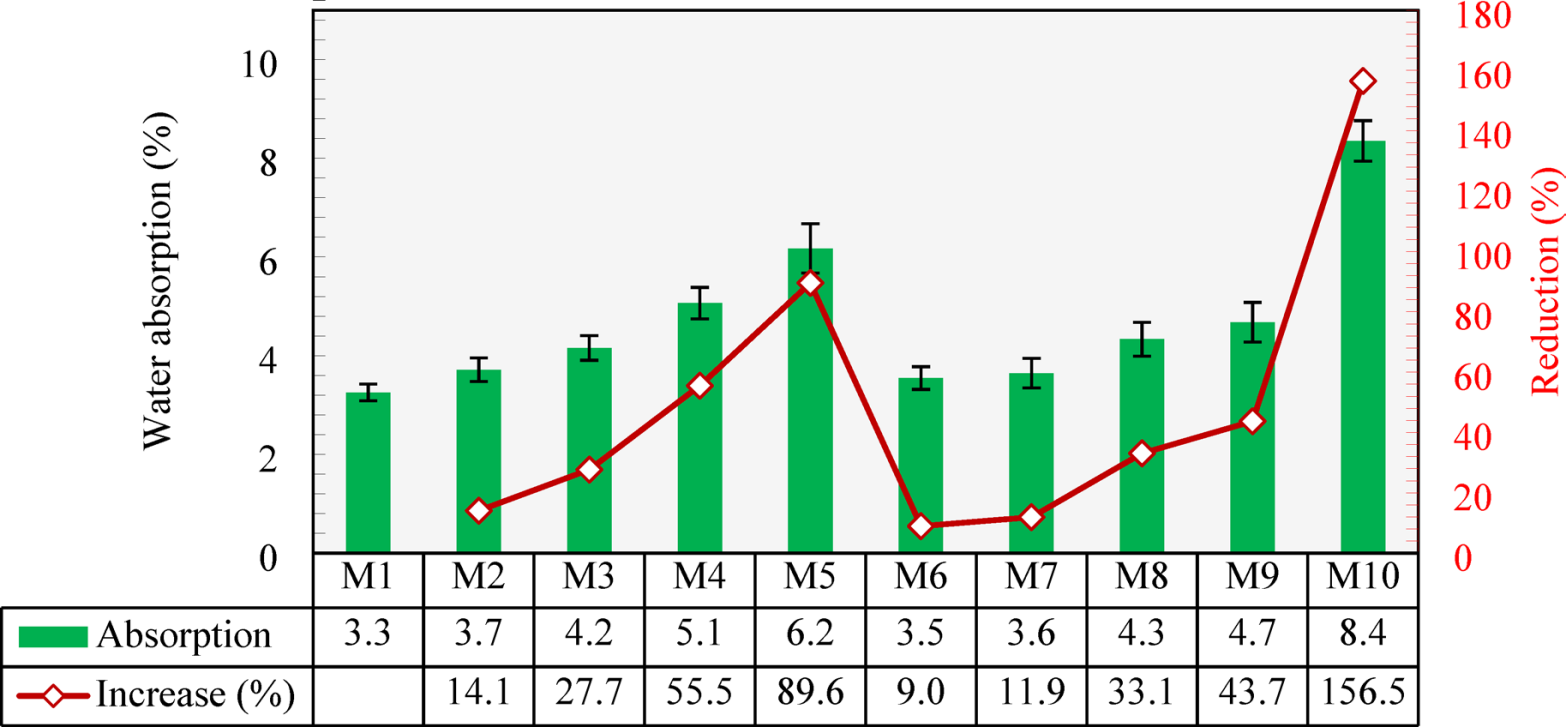
Water absorption of all mixtures and percentage reduction compared to the control mixture (M1).

The water absorption capacity measured for all developed mixtures are shown in Fig. 11. The control mixture (M1) exhibited a water absorption capacity of 3.3%. As the NCA was replaced with CCCA, the water absorption increased. Specifically, when 25% and 50% of the NCA were replaced by CCCA (M2 and M3), the absorption increased by 14.1% and27.7%, respectively, compared to M1. With further replacements, more significant increases were observed. The water absorption increased by 55.5% and 89.6% when 75% and 100% CCCA replacements, respectively, were used. This increase could be attributed to the highly porous structure of CCCA, which provides more voids for water retention, thereby resulting in a higher overall water absorption capacity. In addition, since CCCA is extracted from clay-based bricks, it retains the inherent hydrophilic properties of clay—its ability to attract and bond with water molecules easily. This increases the ability of mixtures containing CCCA to absorb more water.

For the same reason, mixtures with CCFA replacements had a higher absorption capacity than that of the control mixture. As can be seen in Fig. 11. M6, M7, M8, and M9 with 25%, 50%, 75%, and 100% CCFA replacements, respectively, exhibited a water absorption capacity of 9%, 11.9%, 33.1%, and 43.7%, respectively, higher than that of M1. However, mixtures with CCFA demonstrated lower absorption values than those demonstrated by mixtures with CCCA. This can be explained by the fact that both aggregates share the hydrophilic properties of clay, but CCCA has a higher void content than CCFA, providing more space for water absorption. When both CCCA and CCFA were included in mixture M10, the water absorption capacity was 156.5% greater than that of M1.

### Sorptivity

**Fig. 12:**
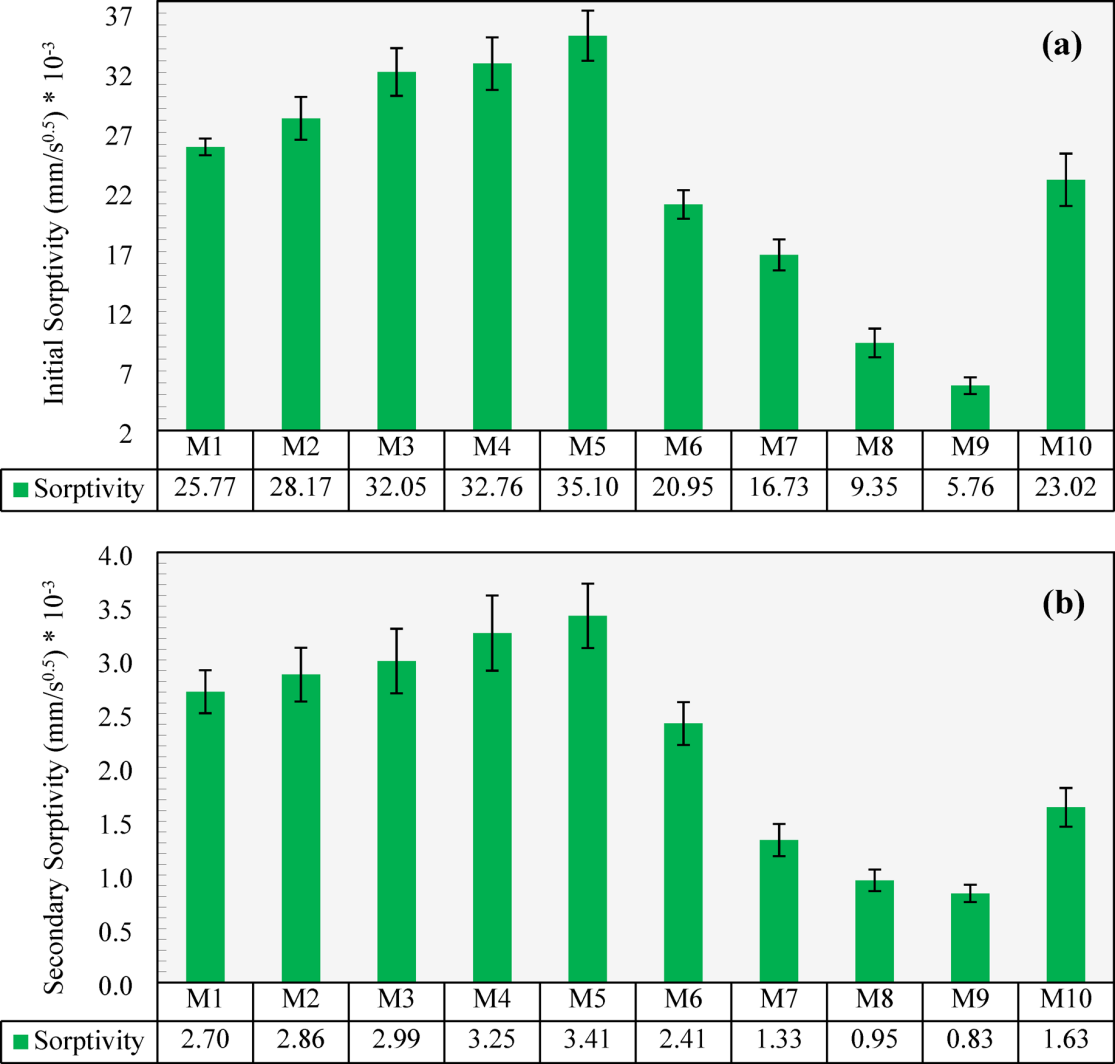
The water sorptivity results of all developed mixtures: (a) initial sorptivity, (b) secondary sorptivity.

In the sorptivity test, capillary suction within the pore system plays a more significant role in water transport in the unsaturated specimens than the material’s permeability and diffusion characteristics^53^. The initial and secondary sorptivity results are presented in Fig. 12. As shown in the figure, M1 (which had no CCCA or CCFA replacements) exhibited initial and secondary sorptivity values of 25.77 × 10⁻³ mm/s⁰.⁵ and 2.7 × 10⁻³ mm/s⁰.⁵, respectively. These values increased when CCCA introduced into the concrete, as M2, M3, and M4 demonstrated a 9.3%, 24.4%, and 27.1% increase in initial sorptivity, respectively, and a 5.9%, 10.6%, and 20.2% increase in secondary sorptivity, respectively. When 100% of the NCA was replaced with CCCA in M5, the initial and secondary sorptivity values increased by 36.2% and 26.1%, respectively, compared to M1, reaching the highest values among all mixes. This increase can be attributed to the greater number of available pores in the CCCA particles’ porous structure, which enhances water retention through capillary suction.

In contrast, the CCFA mixes showed a decrease in sorptivity. For M6 (with 25% CCFA), the initial and secondary sorptivity decreased by 18.7% and 10.9%, respectively, compared to M1. The reductions were more pronounced in M7, M8, and M9 compared to M1, where the initial sorptivity decreased by 35.1%, 63.7%, and 77.6%, respectively, and the secondary sorptivity decreased by 51%, 64.9%, and 69.4%. This reduction may be attributed to the likely denser structure formed by the finer CCFA particles, which have a relatively finer gradation compared to NS, potentially improving packing and restricting water transport through capillary action. The possible beneficial effects of CCFA in forming a denser matrix and reducing sorptivity have also been suggested by other researchers^16,19,20^. It is worth noting that the results of sorptivity do not align with water absorption for CCFA, as explained earlier. This discrepancy arises because, in the water absorption test, specimens are fully submerged, allowing the hydrophilic properties of CCFA, as a clay-based material, to dominate and increase the absorption of concrete. In contrast, during the sorptivity test, the water level covers only the lower portion (about 5 mm to 10 mm) of the specimen. As a result, the microstructure and capillary pores become key factors. Therefore, the finer gradation of CCFA (compared to NS) likely plays a significant role in reducing sorptivity.

When all aggregates were replaced with CCCA and CCFA in M10, the negative impact of CCCA was partly offset by the benefits of the CCFA replacement. As a result, the initial and secondary sorptivity values decreased by 10.7% and 39.8%, respectively, compared to M1.

### Compressive strength

**Fig. 13:**
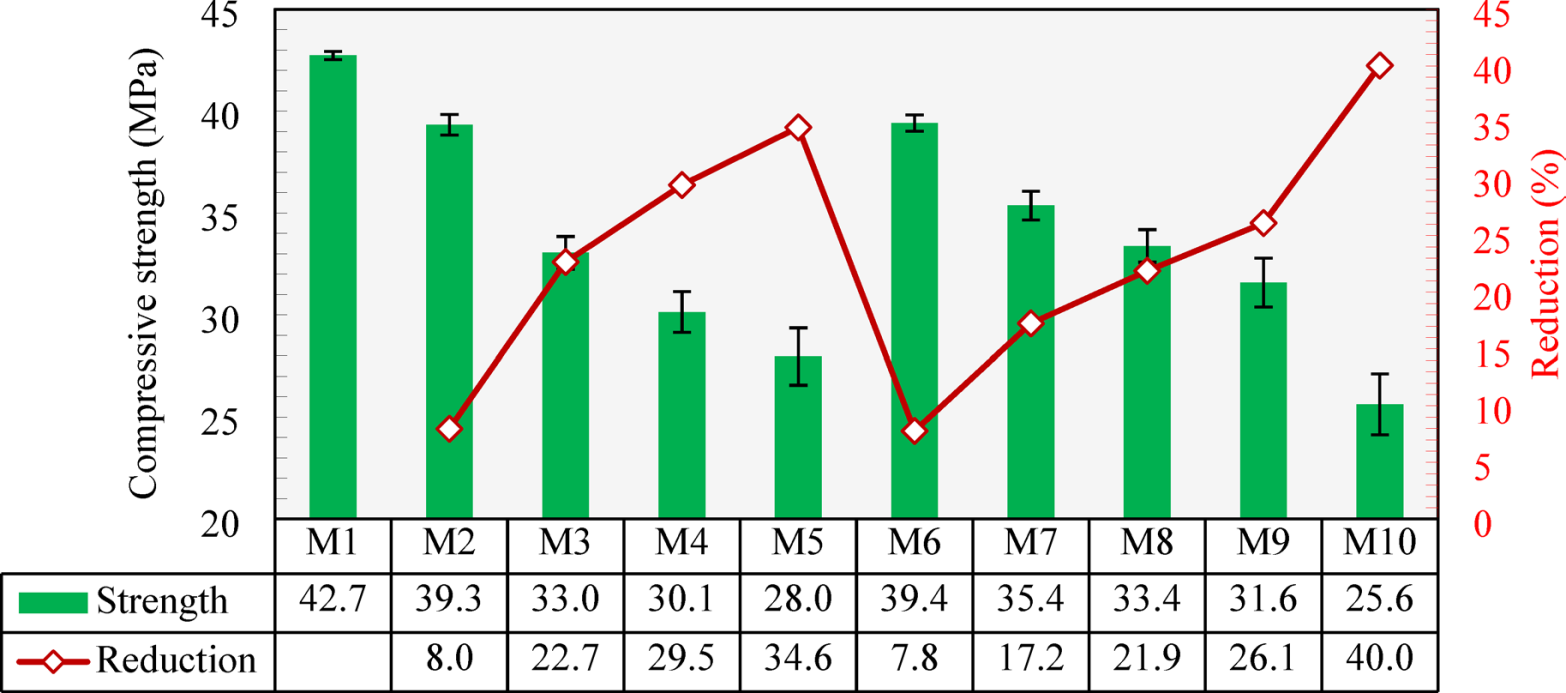
Compressive strength of all developed mixtures and percentage reduction compared to the control mixture (M1).

The compressive strengths of all developed mixtures are presented in Fig. 13. As shown, the control mixture (Mix 1) had a compressive strength of 42.7 MPa. Replacing NCA with CCCA generally led to a reduction in compressive strength. When 25% of the NCA was replaced with CCCA, the compressive strength decreased by 8.0% compared to M1. For the 50% and 75% replacements (M3 and M4), the strength reductions were 22.6% and 29.4%, respectively. The greatest reduction occurred at the 100% replacement (M5), where the strength dropped by 34.5% compared to M1. These findings align with those of Atyia et al.^31^, who reported up to a 33% reduction in compressive strength at full CCCA replacement. Other researchers have documented even greater reductions: for instance^30^, reported losses ranging from 16 to 63% corresponding to 25–100% CCCA replacement levels, while Zhang et al.^29^ observed an 18.3% drop in compressive strength at just 30% replacement. In contrast, Zheng et al.^24^ reported relatively lower reductions, ranging from 3.3 to 13% at 25–100% replacement levels. These reductions were due to the weaker and porous structure of CCCA compared to NCA. The weaker strength of CCCA was also proved by the high crushing index of CCCA, listed in Table 1, which is 2.67 times greater than that of NCA. Additionally, the mechanical crushing used to process CCCA can induce cracks in aggregate particles, resulting in additional weak points within the concrete composite.

Figure 13 also illustrates a decline in compressive strength when NS was replaced with CCFA. With 25%, 50%, 75%, and 100% CCFA replacement (M6, M7, M8, and M9), the compressive strength decreased by 7.7%, 17.2%, 21.8%, and 26.0%, respectively, compared to M1. Higher reductions were reported by^33^ who observed a 49% reduction when 100% CCFA was used. These reductions reflect how the inclusion of weaker fine aggregate (CCFA compared to NS) negatively impacted the mortar’s strength, consequently reducing the overall strength of the concrete. Although both CCCA and CCFA replacements negatively affected the compressive strength, the use of CCFA appeared to have less detrimental effects at the same replacement level. This suggests that, despite both materials being weaker than natural aggregates, the finer and less porous nature of CCFA likely contributes to a better composite, while CCCA’s more porous structure seems to cause a greater reduction in compressive strength.

The M10 mixture, fully developed with CCCA and CCFA, displayed the lowest compressive strength at 25.6 MPa, which is a 40% reduction compared to M1. Despite this, it still surpassed the minimum compressive strength requirement of 17 MPa and 20 MPa for structural concrete outlined in ACI 318^59^ and CSA^57^ as well as meets the minimum criteria for C20/25 structural concrete as per EN 1992-1-1^60^. Furthermore, as previously mentioned, M10 can be categorized as semi-lightweight concrete, making it appropriate for various lightweight structures.

### Splitting tensile strength

**Fig. 14:**
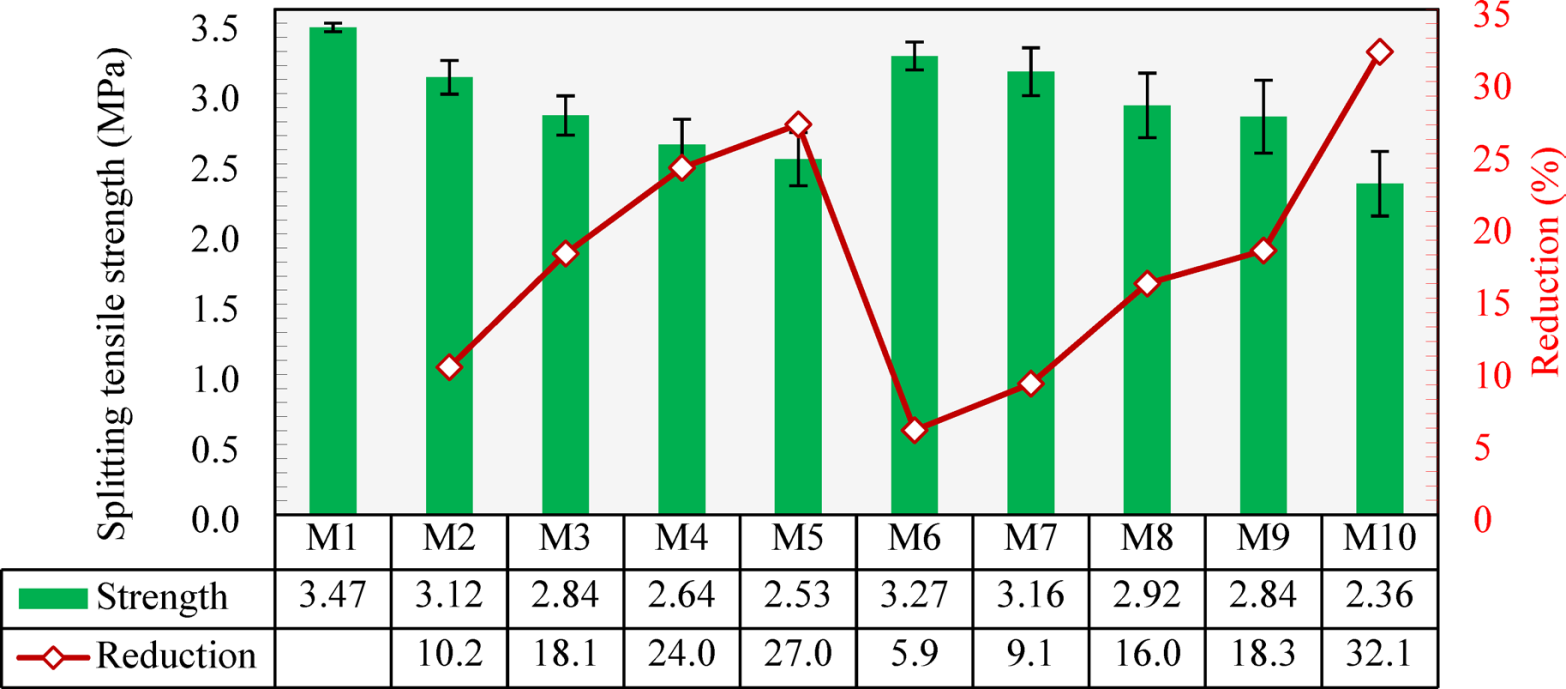
Splitting tensile strength of all developed mixtures and percentage reduction compared to the control mixture (M1).

The splitting tensile strengths of the developed mixtures are presented in Fig. 14. Mixture M1, exhibited a splitting tensile strength of 3.47 MPa. Similar to the compressive strength results, the splitting tensile strength gradually decreased with the inclusion of CCCA. Specifically, M2 and M3, with 25% and 50% CCCA replacements, showed reductions of 10.1% and 18.1%, respectively, compared to M1. For mixtures M4 and M5, with 75% and 100% CCCA, the reductions were more pronounced, reached values of 24% and 27%, respectively. These reductions could be attributed to that the weak strength and porous structure of the CCCA may provide an ideal medium for the initiation and propagation of tensile cracks, which limited the ultimate tensile strength of concrete.

The reductions in splitting tensile strength were smaller when CCFA was used. Mixtures M6, M7, M8, and M9 showed reductions of 5.9%, 9.1%, 16%, and 18.3%, respectively, compared to M1. These results indicate that CCFA has a less negative effect on the tensile strength of concrete than CCCA, similar to what observed in the compressive strength results. It is worth noting that the reductions observed in both CCCA and CCFA mixtures align with the trends reported in previous studies^29,33^, though the magnitudes are significantly lower. This discrepancy may be attributed to differences in mixture composition and the types of materials used, which influence the performance of both aggregates.

When both CCCA and CCFA were used in equal replacement (100%), as in M10, the splitting tensile strength significantly decreased, reaching two-thirds of the original strength at 2.36 MPa (i.e., 32.1% reduction). This substantial drop is primarily due to the combined effects of the weak coarse aggregate and the weakened mortar caused by the fine aggregate.

According to ACI 318^59^, the splitting tensile strength is estimated based on Eq. 3.

3 $$\:STS=\:0.56\sqrt{{f}_{c}^{{\prime\:}}}$$

where $\:STS$ is the splitting tensile strength and $\:{f}_{c}^{{\prime\:}}$ is the compressive strength.

**Fig. 15:**
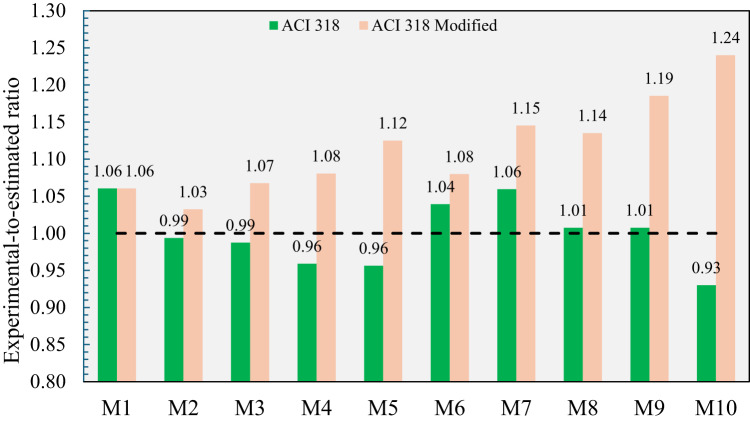
Experimental-to-estimated splitting tensile strength ratios of all developed mixtures.

As depicted in Fig. 15, M1 made with normal aggregates exhibited a splitting tensile strength in a good agreement with that estimated by ACI 318’s equation. On the other hand, as the content of CCCA or CCFA increased, the experimental-to-estimated ratio generally decreased, suggesting that Eq. 3 was unable to accurately capture the effect of including CCCA or CCFA on concrete’s tensile strength. This indicates that relying solely on deriving the splitting tensile strength from the corresponding compressive strength may not be a reliable approach when using such special types of aggregates. The lowest experimental-to-estimated ratio, 0.93, was observed in M10, where both CCCA and CCFA were incorporated. Although CCCA and CCFA are not categorized as lightweight aggregates based on their measured specific gravity, their inherent weakness and porous structure significantly decayed the tensile strength of the concrete. To address this discrepancy, modified estimations were derived by applying the λ factor, as recommended by ACI 318 for lightweight aggregates. Specifically, λ was set to 1 for normal concrete (M1), 0.85 for concrete made entirely of either CCCA or CCFA (M5 and M9), and 0.75 for concrete composed entirely of both CCCA and CCFA (M10). For intermediate mixes (M2, M3, M4, M6, M7, and M8), linear interpolation between 0.85 and 1.0 was applied based on the replacement level. This adjustment yielded more conservative estimations, with the experimental-to-predicted ratios falling within the range of 1.03 to 1.24. However, it is important to note that these findings are based on a limited set of experimental data, and further studies are required to validate these results comprehensively. In addition, as a larger dataset for concrete made with recycled crushed clay aggregates emerges, an independent data-driven model could be developed for more accurate predictions.

### Flexural strength

**Fig. 16:**
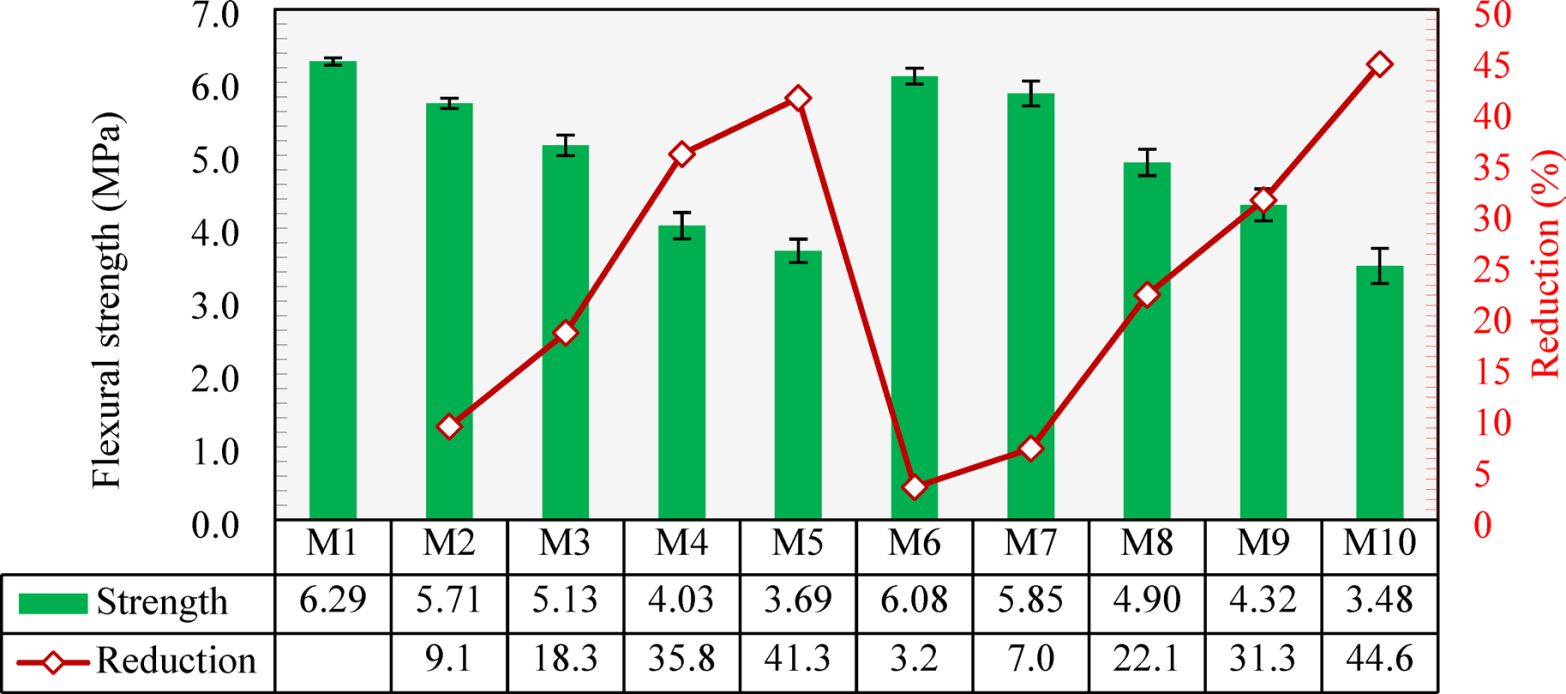
Flexural strength of all developed mixtures and percentage reduction compared to the control mixture (M1).

Figure 16 shows the flexural strengths of all the developed mixtures. The control mixture (M1) achieved the highest flexural strength of 6.29 MPa. For the mixtures containing CCCA, the flexural strength showed a gradual decrease as the replacement increased. In M2 and M3, which incorporated 25% and 50% of the NCA replaced with CCCA, showed reductions in flexural strength of 9.1% and 18.3%, respectively. As the content of high porous and weaker CCCA (compared to NCA) replacement increased, the decrease in flexural strength became more significant. Mixtures M4 and M5, with 75% and 100% CCCA replacement, exhibited reductions of 35.8% and 41.3%, respectively. These reductions are in full agreement with the findings of^30^, who reported strength losses ranging from 17 to 40% when CCCA was used at replacement levels between 25% and 100%.

Mixtures incorporating CCFA showed less decreases in the flexural strength. For M6 and M7, which contained 25% and 50% replacement of fine aggregate with CCFA, the flexural strength decreased by only 3.2% and 7.0%, respectively, compared to M1. As the CCFA replacement increased, the reductions in flexural strength became more notable. Mixtures M8 and M9, with 75% and 100% CCFA replacement, recorded reductions of 22.1% and 31.3%, respectively, compared to M1. These reduction values further confirm the findings observed in the splitting tensile strength results, which suggested that CCFA has a less aggressive effect on tensile strength compared to CCCA.

Mixture M10, which had 100% replacement of both coarse and fine aggregates with CCCA and CCFA, respectively, demonstrated the lowest flexural strength at 3.48 MPa, which represents a reduction of 44.6% compared to M1.

As per ACI 318^59^, the flexural strength (*FS*) is estimated based on Eq. 4.

4 $$\:FS=\:0.62\sqrt{{f}_{c}^{{\prime\:}}}$$

**Fig. 17:**
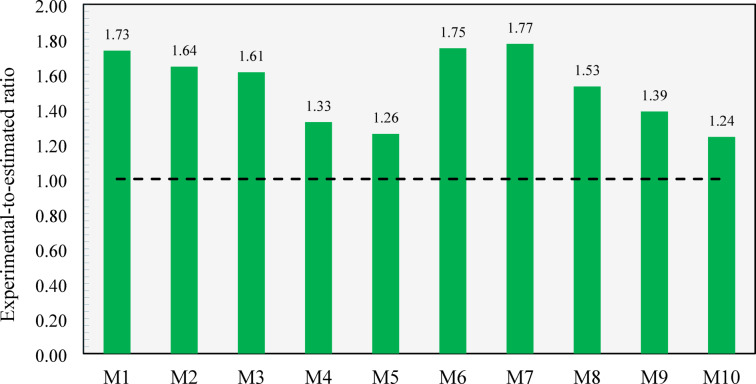
Experimental-to-estimated flexural strength ratios of all developed mixtures.

As depicted in Fig. 17, the ACI 318 equation provided conservative estimates for the flexural strength of all the developed mixtures. However, the conservatism of the equation diminished as the replacement level of CCCA or CCFA increased, with the lowest experimental-to-estimated ratio of 1.24 observed when 100% CCCA and 100% CCFA were combined in M10. The declining trend in the experimental-to-estimated ratios with higher CCCA and/or CCFA content highlights the necessity for further investigation into the adequacy of the code equation for predicting the flexural strength of concrete made with these special types of aggregates, rather than relying solely on compressive strength as a basis, as also previously concluded for splitting tensile strength equation (Eq. 3).

### Abrasion resistance

**Fig. 18:**
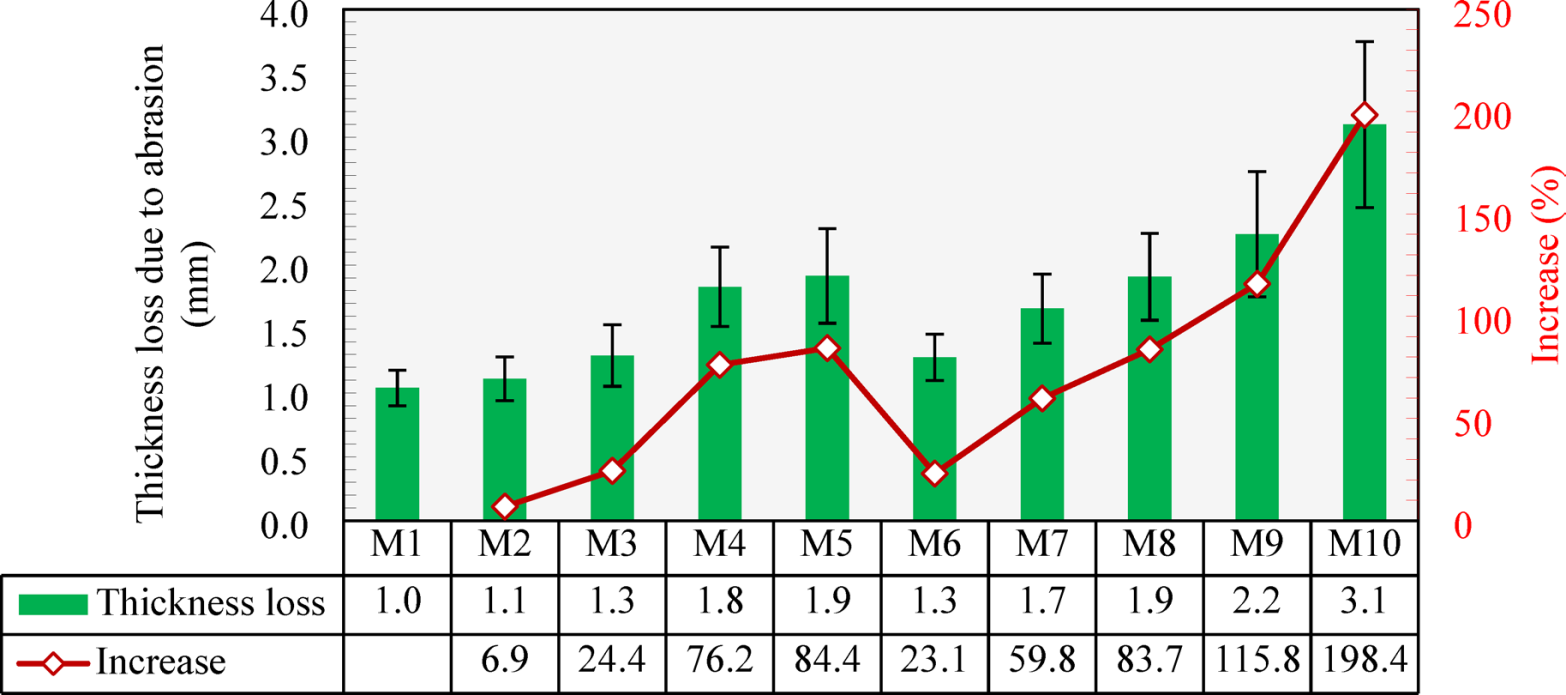
Abraded thickness of all tested specimens and percentage increase compared to the control mixture (M1).

Figure 18 shows the abraded thickness measured for all tested specimens. As can be seen from the figure, the control mix (M1) had 1.0 mm loss in thickness, and as the content of CCCA increased, the abrasion resistance decreased, showing larger abraded thickness. The 25% and 50% CCCA replacements (M2 and M3, respectively) led to 6.9% and 24.4% increase in the abraded thickness compared to M1. Higher reductions in the abrasion resistance were observed when CCCA was used at replacement levels of 75% and 100% in M4 and M5, reaching abraded thickness of 76.2% and 84.4%, respectively, higher than that of M1. The reduction in abrasion resistance is due to the weaker and less dense structure of CCCA particles, which makes them more easily abraded. Subsequently, as the CCCA content increases, there is greater material loss.

Figure 18 also shows the effect of using CCFA on abrasion resistance. The results indicated that CCFA had more significant reductions in abrasion resistance (i.e., yielded larger abraded thickness) compared to CCCA. For instance, M6 with 25% CCFA replacement, lost 23.1% more thickness than the control mix (M1). As the replacement level increased to 50%, 75%, and 100%, the thickness loss increased significantly, reaching 59.8%, 83.7%, and 115.8%, respectively. These higher values of thickness loss, compared to those yielded by CCCA, could be attributed to the weakened mortar caused by the inclusion of CCFA, which governs the surface layers typically exposed to abrasive material.

M10 showed the highest abraded thickness reaching a value of 3.1 mm, which was 198.4% greater than that of M1. This significant loss occurred because the use of CCCA and CCFA diminished the contribution of both mortar and coarse aggregates to abrasion resistance, resulting in a weak composite that was more easily abraded.

### Impact resistance of the cylindrical specimens

**Fig. 19:**
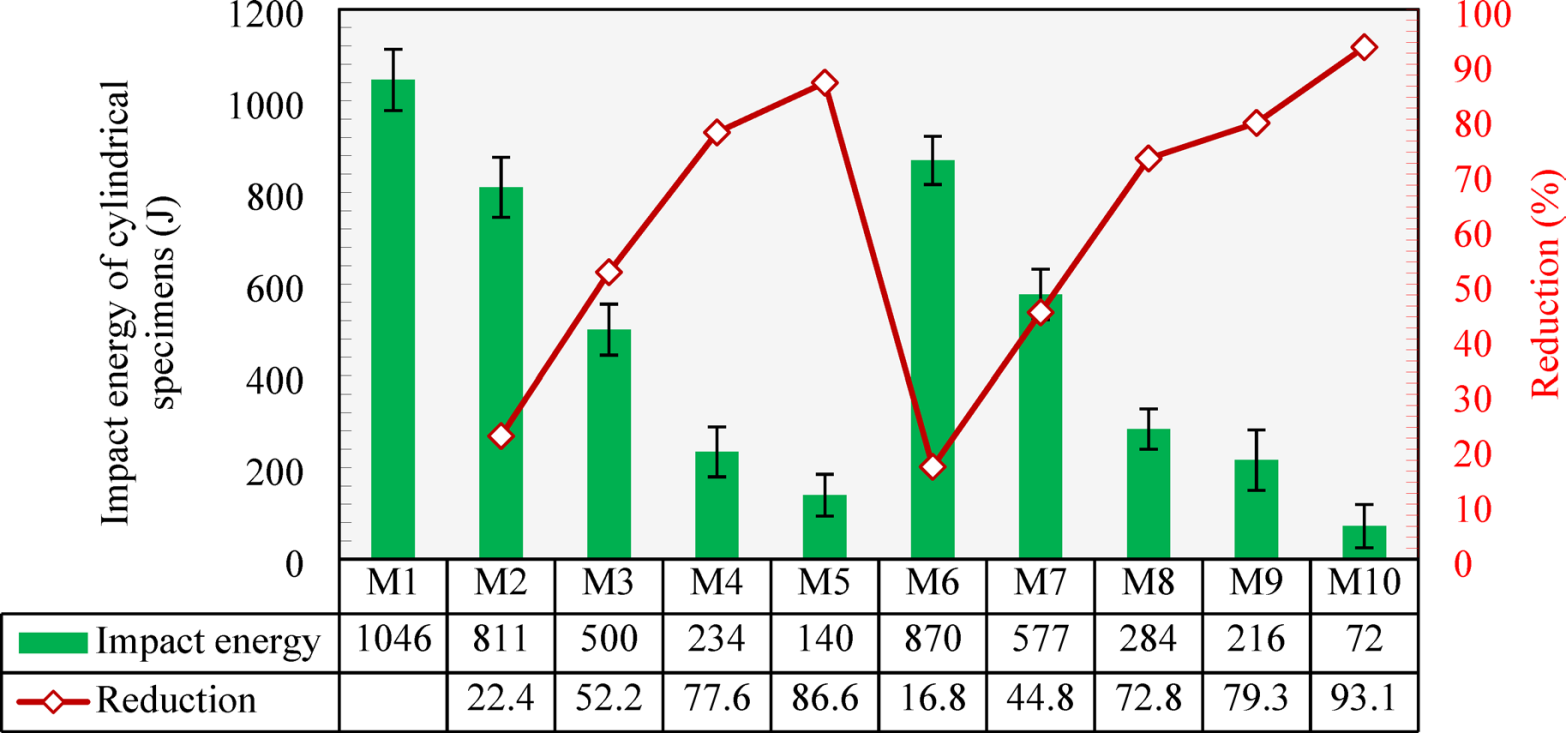
The Impact energy absorption of all developed mixtures (cylindrical specimens) and percentage reduction compared to the control mixture (M1).

The impact resistance results of all developed mixtures obtained from testing the cylindrical specimens are illustrated in Fig. 19. It was found that the failure in the specimens of the control mixture (M1) was induced by impact energy of 1046 J. Lower impact energy by 22.4% was required to break specimens cast with M2 (incorporating 25% of CCCA). Further CCCA content led to a significant decay in the impact energy, reaching reductions of 52.2%, 77.6% and 86.6% for M3, M4 and M5 (50%, 75% and 100% CCCA), respectively, compared to M1. These noticeable deteriorations reflect how weak coarse aggregates (CCCA) can limit the energy absorption capacity of concrete.

As the mortar was weakened when NS was replaced with CCFA, the impact resistance was reduced in a similar trend to CCCA mixtures but with better performance. As shown in Fig. 19, M6, M7, M8, and M9, which had 25%, 50%, 75%, and 100% CCFA replacement, exhibited 16.8%, 44.8%, 72.8%, and 79.3% reductions in the absorbed energy compared to that of the M1.

After replacing 100% of both aggregates in M10, an aggressive reduction of 93.1% was recorded. The energy absorbed caused failure was only 72 J representing only 7% of that absorbed by M1.

### Flexural impact resistance

**Fig. 20:**
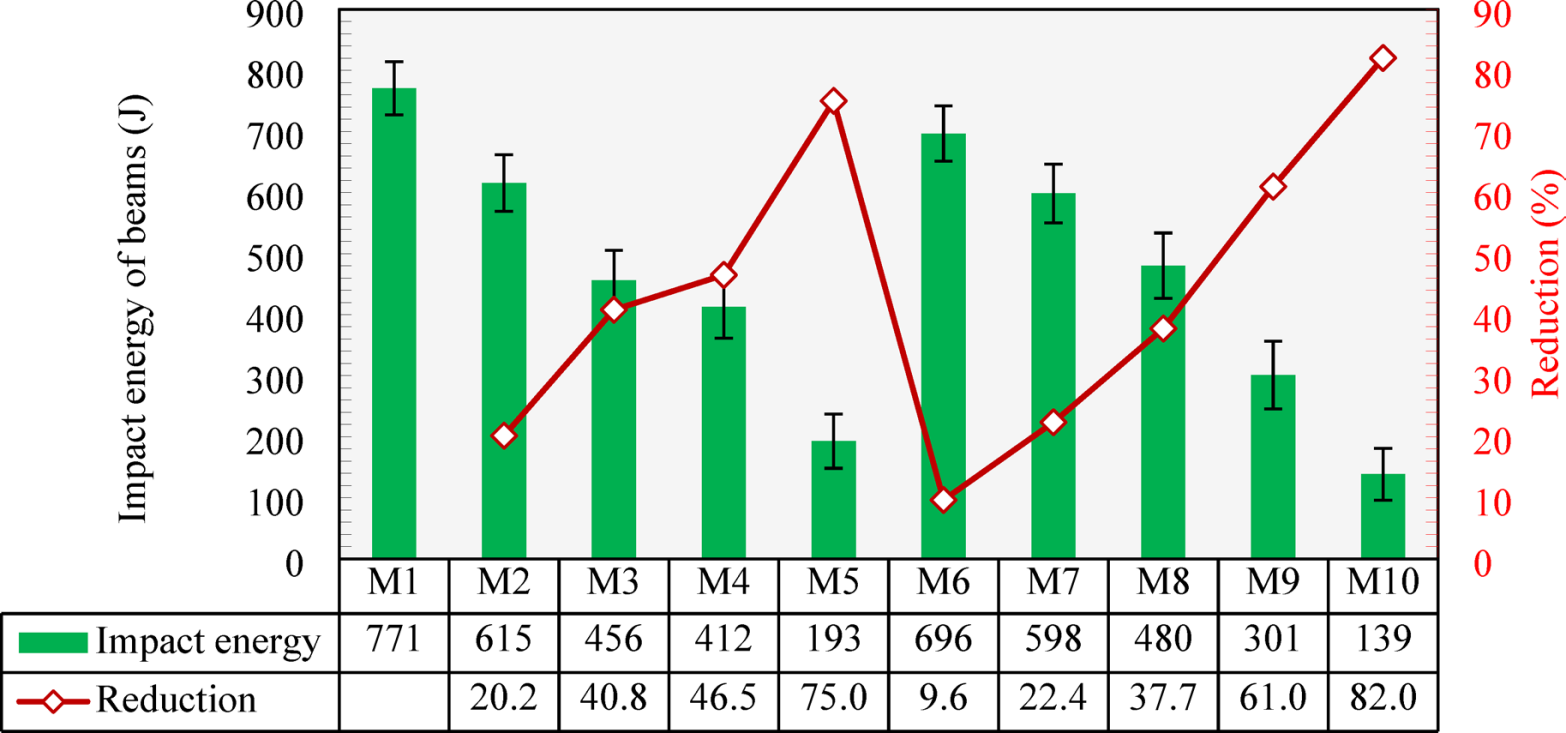
Impact energy of all tested beams and percentage reduction compared to the control mixture (M1).

Figure 20 presents the impact energy results of the tested beams. As can be seen, the results of beams followed a similar response shown by cylindrical specimens, where the impact energy was reduced with the increase of either CCCA or CCFA content. Compared to the maximum impact energy exhibited by M1 (771 J), the use of 25%, 50%, and 75% CCCA in M2, M3, and M4 led to 20.2%, 40.8%, and 46.5% reductions. The highest reduction in the absorbed energy, 75%, was demonstrated by M5 (with 100% CCCA), reaching a value of 193 J.

Similar to the cylindrical specimens, the use of CCFA showed better results under impact loading. With 25%, 50%, and 75% CCFA replacement levels, mixtures M6, M7, and M8, respectively, exhibited reductions in the absorbed energy of 9.7%, 22.4%, and 37.7%, respectively, compared to M1. This reduction reached 61% in M9 (100% CCFA) when compared to M1. The lowest impact resistance was recorded by M10, which failed at an impact energy of 139 J, that is 82% less than that of M1.

### Ultrasonic pulse velocity (UPV)

**Fig. 21:**
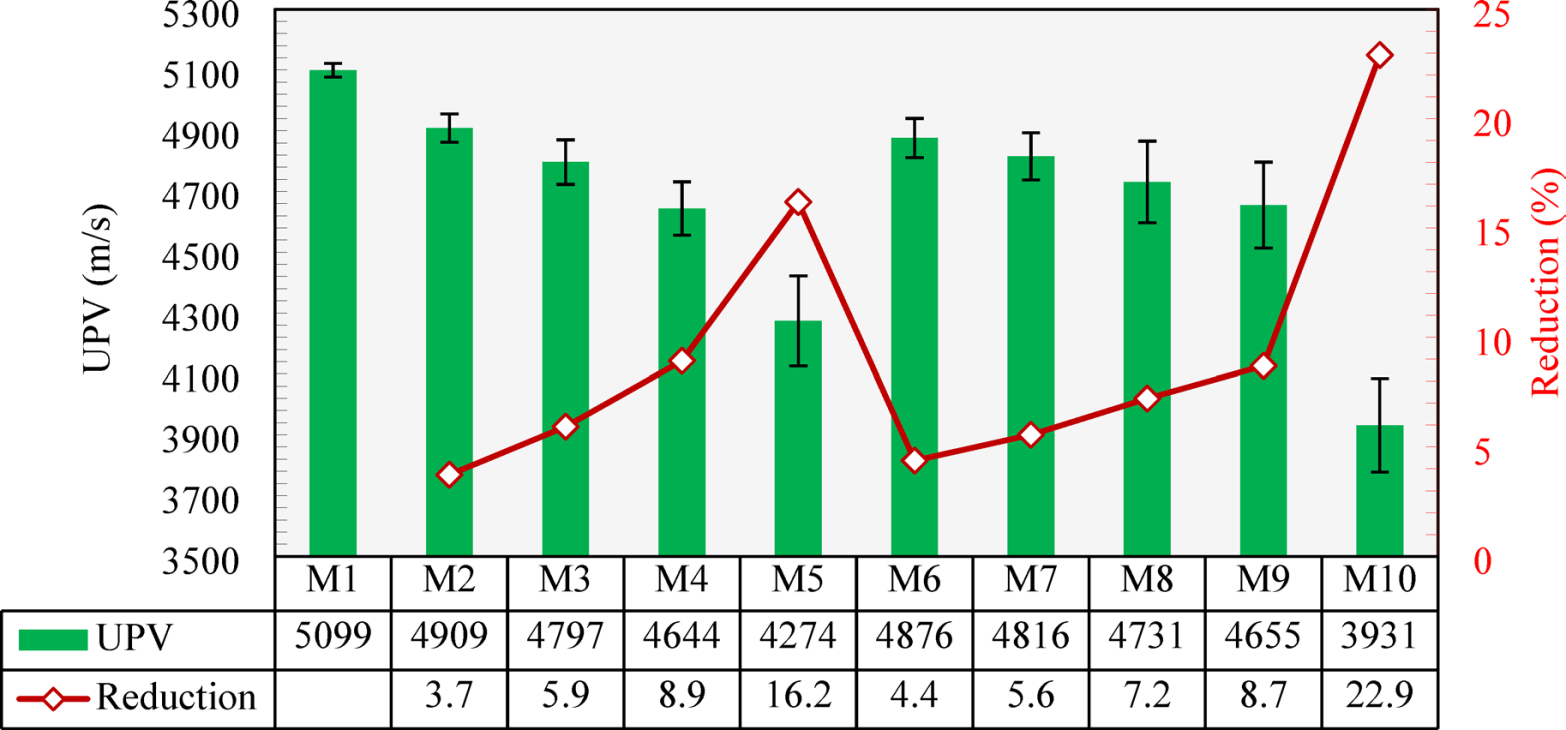
Variation of UPV across mixtures and percentage reductioncompared to the control mixture (M1).

The UPV results measured for all developed mixtures are displayed in Fig. 21. As shown, the control mix (M1) exhibited a value of 5099 m/s. Inclusion of the CCCA into the concrete instead of the NCA caused a decrease in the UPV. Compared to M1, reductions of 3.7%, 5.9%, and 8.9% in the UPV were recorded when 25%, 50%, and 75% of CCCA (M2, M3, and M4) were used. M5, with 100% CCCA replacement, showed a higher reduction of 16.2% compared to that of M1. The main reason for this was the porosity of the CCCA, which contributed to attenuating the velocity of ultrasonic waves.

Mixtures with CCFA also showed less UPV values. The inclusion of 25%, 50%, 75%, and 100% CCFA replacements exhibited 4.4%, 5.6%, 7.2%, and 8.7% reductions in the UPV, respectively, compared to M1. Although CCFA replacements caused a reduction in UPV, they had less effects compared to CCCA replacements, especially in high replacements beyond 50%. This could be related to the higher voids content in CCCA compared to the finer CCFA particles.

When all fine and coarse aggregates were replaced by the CCCA and CCFA, a maximum reduction of 22.9% was observed in UPV, demonstrating a value of 3931 m/s. As per CBD-187^61^ and IS 13,311^62^, all mixtures achieved “excellent concrete quality” as their UPV is higher than 4500 m/s except for M5 and M10 which are considered “good concrete quality” as they passed 3500 m/s in the UPV test.

**Fig. 22:**
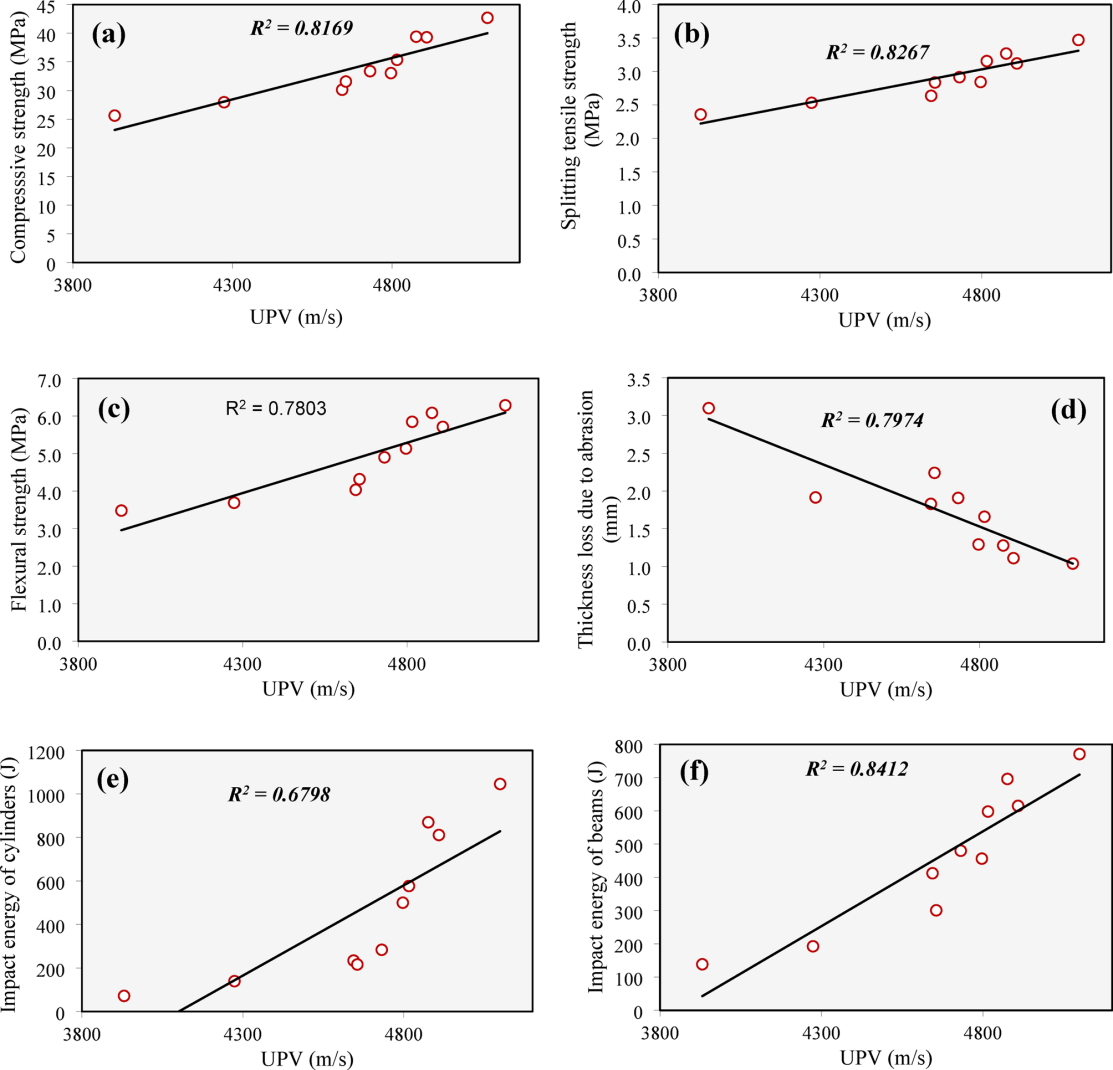
Correlation between UPV and mechanical properties: (**a**) Compressive strength, (**b**) splitting tensile strength, (**c**) flexural strength, (**d**) thickness loss due to abrasion, (**e**) impact energy of cylinders, (**f**) impact energy of beams.

Figure 22 presents the relationship between UPV and all measured mechanical properties, including compressive strength, splitting tensile strength, flexural strength, abrasion resistance, and impact resistance. In general, a decline in UPV—which indicates a higher presence of defects such as cracks, and voids—was linked to a decrease in all the investigated mechanical properties.

Despite these general trends, the degree of correlation between UPV and the mechanical properties varied significantly across different properties. The strongest correlation was found between UPV and the impact energy of beams, with a high correlation coefficient (*R²* = 0.8619). On the other hand, the relationship between UPV and the impact energy of cylindrical specimens was the weakest, as evidenced by a lower correlation coefficient (*R²* = 0.6798).

### Schmidt hammer

**Fig. 23:**
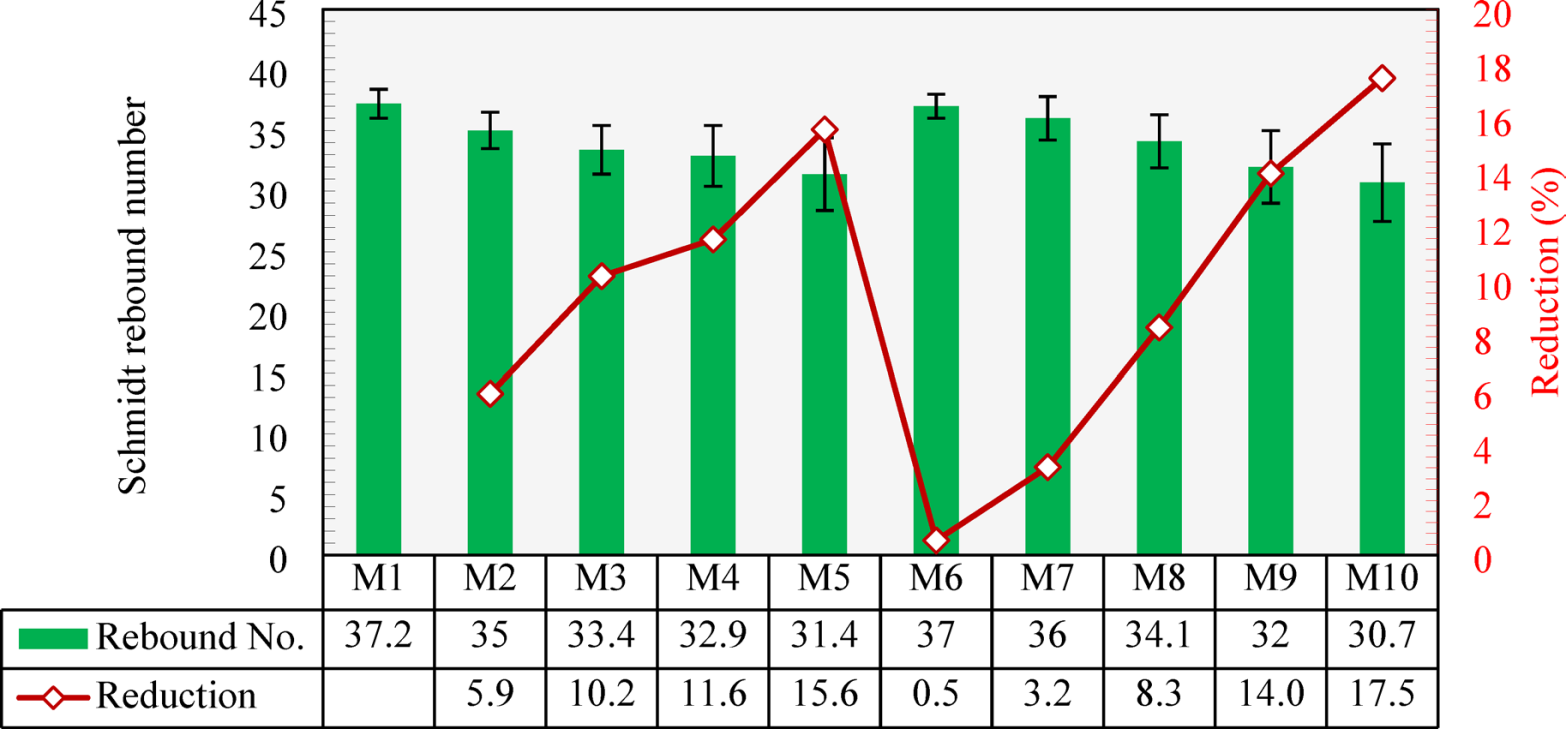
Schmidt rebound number of all mixtures and percentage reduction compared to the control mixture (M1).

Figure 23 shows the results of the Schmidt hammer test. As can be seen, M1 had an average rebound number of 37.2. Replacing 25%, 50%, and 75% of the NCA by the CCCA in M2, M3, and M4 resulted in reductions in the Schmidt hammer reading by 5.9%, 10.2%, and 11.6%, respectively, compared to that of M1. M5, which had a 100% CCCA replacement, had a higher reduction of 15.6%. This decrease in rebound readings due to the low stiffness of CCCA compared to NCA, which in turn reduced the overall stiffness of concrete composite, thus yielding lower rebound numbers.

The Schmidt hammer readings for CCFA mixture (M5-M9) were not affected as much as the CCCA mixtures (M2-M5), especially prior to replacement level of 100%. M6, with 25% CCFA, experienced almost no reduction, while M7 and M8 had relatively lower reductions at 3.2% and 8.3% respectively. The greatest effects were observed in the mixture with 100% CCFA (M9), where the reduction reached up to 14% compared to M1. Such reductions occurred due to the weakening of the mortar stiffness that was caused by the inclusion of the CCFA. However, the effect of CCCA replacements were generally more pronounced due to the porous structure of CCCA which had a more dominant effect on the rebound readings. M10, which had 100% CCCA and 100% CCFA substitutions, recorded the minimum rebound number of 30.7, corresponding to a reduction of 17.5% compared to M1.

**Fig. 24:**
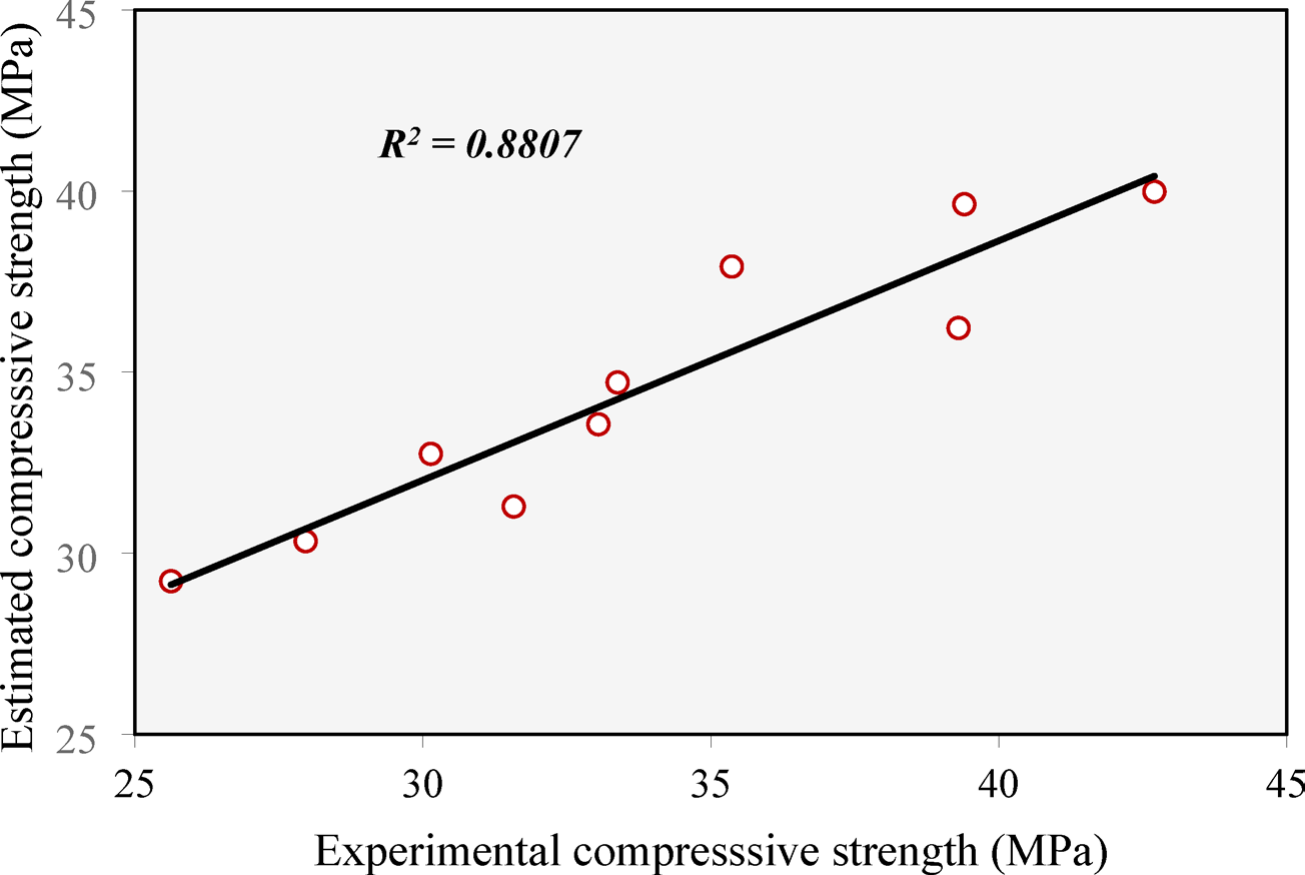
Correlation between the estimated compressive strength by Schmidt hammer test against the experimental compressive strength.

Figure 24 shows correlation between the experimental compressive strength and that estimated by the Schmidt hammer test. It should be noted that the estimated compressive strengths were obtained from the calibration curve provided by the manufacturer. The figure indicates a good correlation between the experimental and the estimated values, achieving *R*^*2*^ of 0.8807. The experimental-to-estimated ratios ranged from 0.88 to 1.09 with an average of 0.98 and a coefficient of variance 6.82%. This indicates that the Schmidt hammer test can be used to evaluate concrete made of CCCA and/or CCFA, however, further calibrations need to be conducted in order to improve accuracy.

**Fig. 25:**
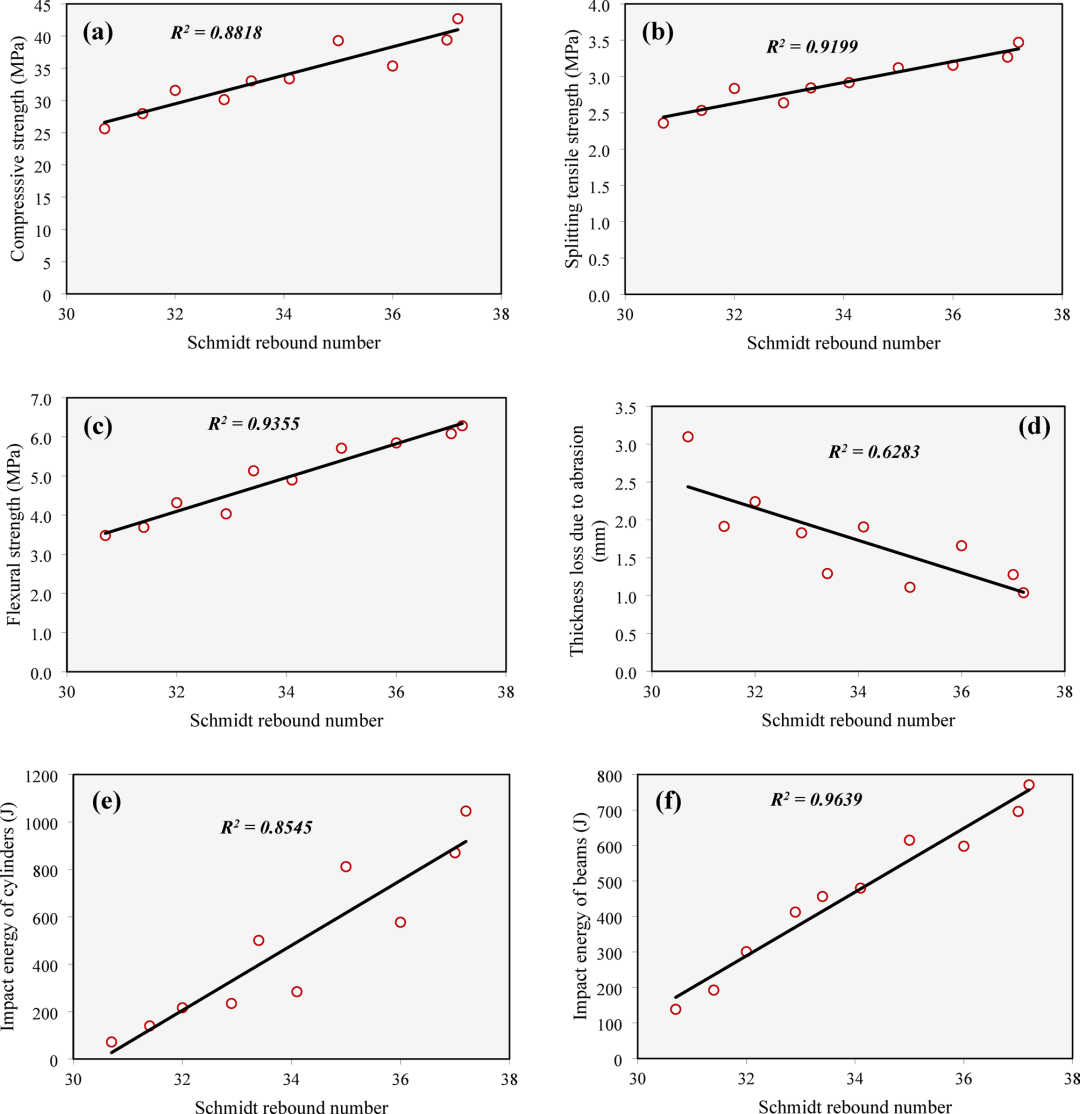
Correlation between Schmidt hammer and mechanical properties: (**a**) Compressive strength, (**b**) splitting tensile strength, (**c**) flexural strength, (**d**) thickness loss due to abrasion, (**e**) impact energy of cylinders, (**f**) impact energy of beams.

Figure 25 illustrates the relationship between the investigated mechanical properties and the rebound number of the Schmidt hammer. In a manner similar to the findings with the UPV, an increase in the rebound number was associated with a rise in compressive strength, splitting tensile strength, flexural strength, and impact energy absorption for both cylinders and beams. Additionally, mixtures with a higher rebound number demonstrated a reduction in the abraded thickness. The figure also highlights that the Schmidt hammer provided stronger correlations with the measured mechanical properties compared to those provided by the UPV. Specifically, the correlation coefficients for the Schmidt hammer ranged from 0.6283 to 0.9639, with the highest correlation observed for flexural impact energy of beams, similar to the UPV results. In contrast, the lowest correlation was found for the thickness loss due to abrasion.

It is important to note that non-destructive testing methods such as the Schmidt hammer and UPV can be valuable tools for indirectly assessing the uniformity and internal integrity of concrete. These techniques provide rapid, non-invasive evaluations of concrete quality; however, their reliability and accuracy can be compromised when recycled aggregates are used. Recycled clay brick aggregates, in particular, tend to exhibit substantial variability in porosity, stiffness, composition, and surface texture, which can result in inconsistent rebound values in the Schmidt hammer test and disrupted wave transmission in the UPV test. Moreover, the process of producing clay brick aggregates—particularly crushing—can introduce additional microcracks and surface damage, further affecting the internal structure of the concrete and complicating the interpretation of non-destructive testing results. As a result, the accuracy of Schmidt hammer and UPV methods in predicting the mechanical performance of concrete containing recycled clay brick aggregate is often limited. To improve their reliability, it is essential to perform further calibrations and develop prediction models specifically tailored to recycled aggregate concrete, taking into account the unique characteristics of the recycled aggregates. Additionally, combining these non-destructive testing methods with complementary testing techniques, such as destructive testing or advanced imaging, may provide a more robust and comprehensive assessment of concrete quality and structural integrity.

## Statistical analysis

### Variability of experimental data

**Fig. 26:**
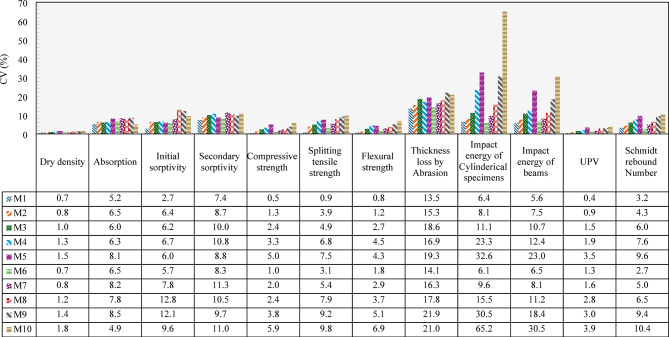
The coefficient of variance for all the experimental data.

All reported experimental results, unless otherwise noted, represent the mean of three independently tested specimens. The error bars in the figures represent the standard deviation, illustrating the spread and consistency of the measured data. To enable clearer comparisons across various mechanical, physical, and transport properties—each expressed in different units—the coefficient of variation (CV) was adopted as a normalized indicator of variability. Figure 26 summarizes the CV values calculated for all concrete mixtures examined in the study (M1–M10). As shown, the control mix M1 consistently exhibited the lowest CV values across all tests, with values of 0.7%, 5.2%, 2.7%, 7.4%, 0.5%, 0.9%, 0.8%, 13.5%, 6.4%, 5.6%, 0.4%, and 3.2% for dry density, water absorption, initial sorptivity, secondary sorptivity, compressive strength, splitting tensile strength, flexural strength, abrasion, impact energy of cylinders, impact energy of beams, UPV, and Schmidt rebound number, respectively. These low CVs reflect minimal dispersion around the mean values, indicating high consistency in performance. In contrast, the inclusion of recycled crushed clay brick aggregates in mixtures M2–M10resulted in comparatively higher CVs, reflecting increased variability in the test results. For M2–M10, CVs ranged from 0.8 to 1.8% for dry density, 4.9–8.5% for water absorption, 5.7–12.8% for initial sorptivity, 8.3–11.3% for secondary sorptivity, 1.0–5.9% for compressive strength, 3.1–9.8% for splitting tensile strength, and 1.2–6.9% for flexural strength. This increase in variability can be attributed to the less uniform nature of recycled clay brick aggregates as manufactured materials compared to natural aggregates. Moreover, the recycling process, particularly crushing, may introduce additional flaws such as microcracks in the particles. These factors contribute to heterogeneities within the concrete matrix, leading to greater variability in the test results. The results also indicated that splitting tensile strength showed higher variability than compressive and flexural strengths, suggesting that tensile strength is more sensitive to inconsistencies caused by recycled materials, particularly due to its dependence on microcracks, aggregate–matrix interface quality, and the presence of voids or defects. Greater variability was observed in the abrasion test, with CVs ranging from 13.5% to 21.9%, highlighting its sensitivity to minor variations in surface conditions. The impact energy of cylindrical specimens and beams showed the highest dispersion, with CVs ranging from 6.1 to 65.2% and 5.6–30.5%, respectively. This high variability is inherent to the drop-weight impact method, which is highly affected by aggregate size, distribution, surface condition, specimen geometry, and microstructural defects—leads to inconsistent responses when subjected to sudden, concentrated loads. UPV results showed moderate variability (CVs of 0.4–3.9%), while Schmidt rebound numbers exhibited CVs ranging from 2.7 to 10.4%. Notably, mixture M10—with 100% replacement of both coarse and fine aggregates using recycled brick aggregates—tended to produce the highest CVs across nearly all tests, underscoring the greater heterogeneity and increased scatter introduced by increasing recycled aggregates.

### Significance of parameters

The ANOVA results presented in Table 4 highlight the statistical significance of the effects of CCCA, CCFA, and their combined interaction on various concrete properties. For most measured parameters—including dry density, absorption, initial sorptivity, compressive strength, splitting tensile strength, flexural strength, abrasion resistance, impact energy (both cylindrical and beam specimens), and ultrasonic pulse velocity (UPV)—both CCCA and CCFA showed statistically significant effects, with very low p-values (typically < 0.0001), indicating strong influence on these properties. Notably, the interaction term (CCCA + CCFA) was also significant for nearly all properties, suggesting that the combined presence of both types of recycled aggregates can lead to synergistic or compounding effects on performance variability. However, for secondary sorptivity, only CCFA and the interaction term were significant, while CCCA alone was not, indicating that fine aggregates play a more dominant role in capillary transport behavior during the later stages. In the case of initial sorptivity, both CCCA and CCFA had significant individual effects; however, their interaction was not significant. This may be due to their opposing influences on initial sorptivity, which likely diminished each other’s overall influence. On the other hand, Schmidt rebound number, a measure of surface hardness, was not significantly affected by CCCA, CCFA, or their interaction, with all p-values well above 0.05, indicating that this property remains relatively insensitive to variations in recycled brick aggregates content.

**Table 4:** ANOVA results for the effect of CCCA or CCFA on the tested properties.

Property	Factor	DF	Sum of Square	Mean Square	F-statistic	*P*-value	Significance
Dry density	CCCA	4	284101.656	71025.414	79.281	< 0.0001	Significant
	CCFA	4	78547.416	19636.854	22.770	< 0.0001	Significant
	CCCA + CCFA	1	432875.760	432875.760	373.398	< 0.0001	Significant
Absorption	CCCA	4	16.260	4.065	26.705	< 0.0001	Significant
	CCFA	4	4.224	1.056	8.393	0.0031	Significant
	CCCA + CCFA	1	39.015	39.015	262.110	< 0.0001	Significant
Initial sorptivity	CCCA	4	167.820	41.955	8.251	0.0033	Significant
	CCFA	4	807.060	201.765	121.121	< 0.0001	Significant
	CCCA + CCFA	1	11.760	11.760	2.949	0.1611	Not significant
Secondary sorptivity	CCCA	4	0.996	0.249	1.758	0.2139	Not significant
	CCFA	4	9.204	2.301	57.069	< 0.0001	Significant
	CCCA + CCFA	1	1.815	1.815	31.510	0.0049	Significant
Compressive strength	CCCA	4	462.204	115.551	99.568	< 0.0001	Significant
	CCFA	4	245.040	61.260	73.655	< 0.0001	Significant
	CCCA + CCFA	1	438.615	438.615	254.772	< 0.0001	Significant
Splitting tensile strength	CCCA	4	1.980	0.495	17.188	0.0002	Significant
	CCFA	4	0.996	0.249	4.672	0.0219	Significant
	CCCA + CCFA	1	1.815	1.815	63.021	0.0014	Significant
Flexural strength	CCCA	4	14.616	3.654	115.341	< 0.0001	Significant
	CCFA	4	8.664	2.166	53.720	< 0.0001	Significant
	CCCA + CCFA	1	11.760	11.760	326.667	< 0.0001	Significant
Thickness loss by Abrasion	CCCA	4	2.004	0.501	4.945	0.0185	Significant
	CCFA	4	2.724	0.681	4.743	0.0209	Significant
	CCCA + CCFA	1	6.615	6.615	87.442	0.0007	Significant
Impact energy of Cylinderical specimens	CCCA	4	1753470.696	438367.674	86.246	< 0.0001	Significant
	CCFA	4	1557487.056	389371.764	78.121	< 0.0001	Significant
	CCCA + CCFA	1	1422137.535	1422137.535	323.664	< 0.0001	Significant
Impact energy of beams	CCCA	4	570210.516	142552.629	43.085	< 0.0001	Significant
	CCFA	4	412796.004	103199.001	28.194	< 0.0001	Significant
	CCCA + CCFA	1	599515.260	599515.260	217.096	0.0001	Significant
UPV	CCCA	4	1161234.240	290308.560	25.626	< 0.0001	Significant
	CCFA	4	344237.904	86059.476	5.892	0.0106	Significant
	CCCA + CCFA	1	2046686.415	2046686.415	112.782	0.0004	Significant
Schmidt rebound Number	CCCA	4	58.704	14.676	2.134	0.1509	Not significant
	CCFA	4	57.936	14.484	2.481	0.1112	Not significant
	CCCA + CCFA	1	63.375	63.375	7.232	0.0547	Not significant

## Embodied energy of the developed mixes

Quantifying the embodied energy of concrete mixtures is crucial for evaluating their environmental performance, particularly when incorporating alternative aggregates. This study specifically aims to assess the energy consumption associated with processing each type of aggregate used in the developed mixes, including NCA, NS, CCCA, and CCFA. By focusing on the energy required for cleaning, crushing, and sieving processes, the study provides a comparative analysis of the embodied energy contributions from both natural and recycled aggregates. This allows for a clearer understanding of the potential trade off between energy savings and recycling practice when substituting traditional aggregates with recycled clay bricks in concrete production. Recycling clay bricks involves only cleaning, crushing, and grading, which require significantly less energy, compared to that used in processing natural stone. In this study, aggregates represent about 67% of concrete volume, highlighting the potential for significant energy savings through alternative materials. Table 5 presents the energy required to process NCA, NS, CCCA, and CCFA including crushing, sieving, and cleaning, as measured in laboratory by authors. As indicated, crushing clay bricks to CCCA consumed energy roughly 1/3 that is required to crushing the NCA. On the other hand, more energy is required for initially cleaning bricks to remove the adhered mortar. Natural sand, when used in its raw form, requires minimal cleaning, which can be considered negligible. It should be noted that the same sieving technique was applied to all aggregates, with an estimated energy consumption of 0.02 MJ/kg.

**Table 5:** Embodied energy in (MJ/kg) of different processes for each aggregate type.

Aggregate type	Cleaning	Crushing	Sieving	Total
NCA	-	0.3	0.02	0.32
NS	-	-		0.02
CCCA	0.02	0.11		0.15
CCFA	0.02	0.26		0.30

**Fig. 27:**
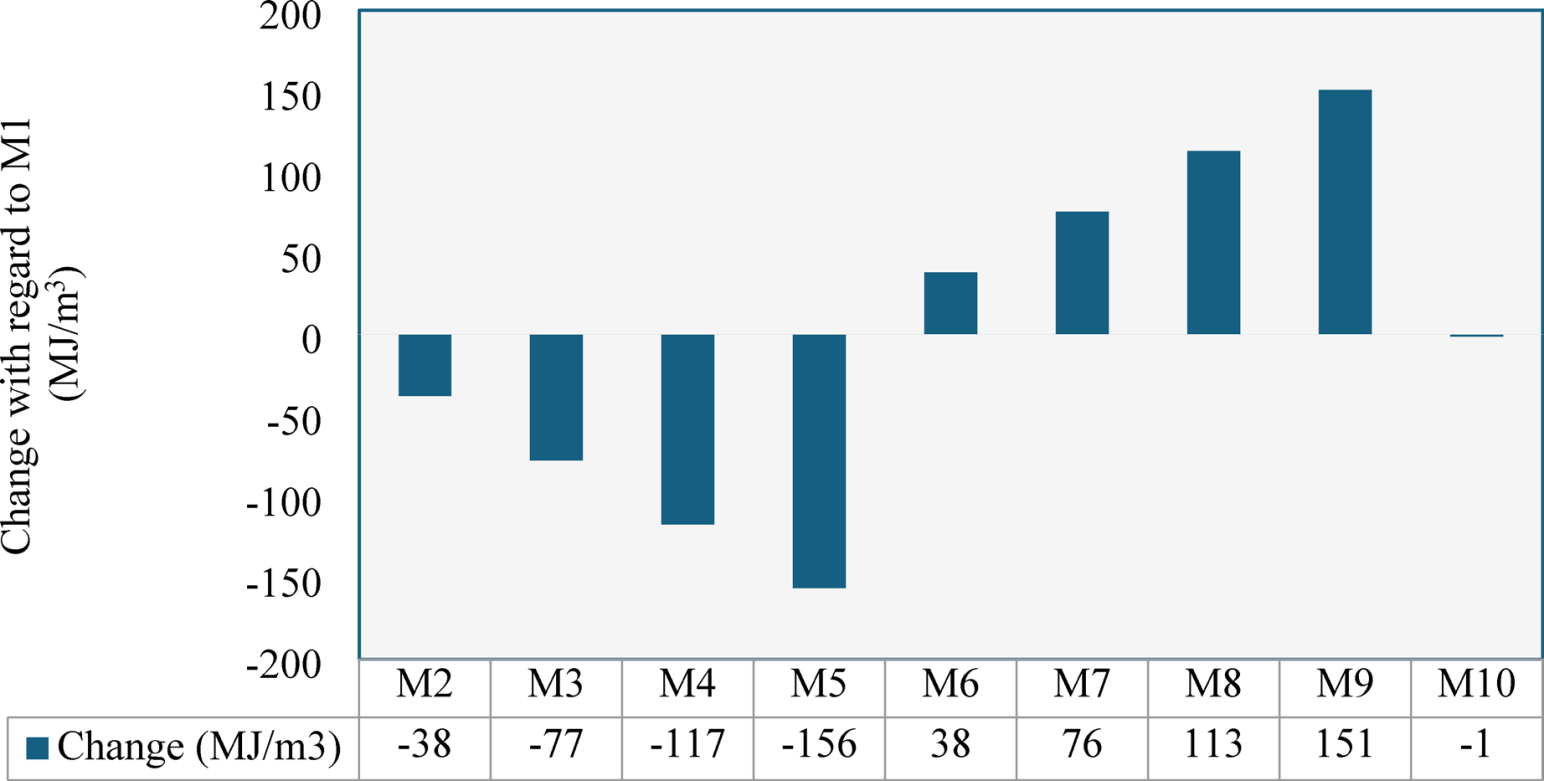
Change in the embodied energy for 1 m3 of each mix with respect to the control mix (M1).

Figure 27 shows how embodied energy per cubic meter varies with different proportions of recycled versus natural aggregates. Replacing NCA with CCCA significantly reduced embodied energy by 13%, 27%, 41%, and 55% at 25%, 50%, 75%, and 100% replacement levels, respectively, compared to the control mix (M1). In contrast, replacing natural sand with CCFA increased embodied energy by 13%, 27%, 40%, and 53% (M6–M9), due to the low initial energy demand of natural sand. Notably, full replacement of both dolomite and sand in mix M10 resulted in an energy footprint nearly equal to the control, demonstrating that complete substitution is feasible without increasing energy demand, while enhancing sustainability through waste reduction and resource conservation. In addition to the energy savings from recycling clay bricks, using them offers further environmental advantages. It helps divert large amounts of non-biodegradable waste from overflowing landfills, which are becoming a growing issue in urban areas. Furthermore, by replacing NS with CCFA, more high-quality sand is preserved for industries where it is essential, such as electronics, solar panel manufacturing, and glass production.

## Conclusions

This study aimed to evaluate the potential of using recycled crushed clay bricks in concrete as a coarse and fine aggregates. Ten mixtures were developed with varying replacement levels of crushed clay coarse aggregate (CCCA) and crushed clay fine aggregate (CCFA), particularly 25%, 50%, 75%, and 100%. The experimental program included a wide range of tests: slump, dry density, water absorption, sorptivity, compressive strength, splitting tensile strength, flexural strength, abrasion resistance, impact resistance (cylindrical and beam specimens), ultrasonic pulse velocity (UPV), and Schmidt hammer. Based on the results, the following conclusions can be drawn:

1. The inclusion of either CCCA or CCFA in concrete negatively impacted its workability, with more significant reductions observed in mixtures containing CCFA at the same replacement level. When both aggregates were combined, the workability decreased by 41% compared to the control mixture (which contained no CCCA or CCFA).
2. The addition of either CCCA or CCFA led to a reduction in dry density, with CCCA having a more significant effect due to its higher void content, which contributed to a lighter weight. Semi-lightweight concrete mixtures, as defined by CSA^58^, could be produced with a density of 2125 kg/m³ when 100% CCCA replacement was used, and a density of 1975 kg/m³ when both CCCA and CCFA were combined.
3. Water absorption increased significantly as the CCCA or CCFA content increased. However, the increase was more pronounced in CCCA mixtures when the replacement level exceeded 25%, while in CCFA mixtures, a significant increase was observed only when the replacement level surpassed 50%.
4. In contrast to water absorption, the sorptivity results showed contradictory behavior. The inclusion of CCCA resulted in an increase in sorptivity, while the mixture containing CCFA exhibited lower sorptivity compared to the control mixture.
5. Increasing the content of CCCA and/or CCFA led to a reduction in the concrete compressive strength. However, CCFA demonstrated better performance, with compressive strength decreasing by up to 34.5% for 100% CCCA replacement and 26% for 100% CCFA replacement. Additionally, it was possible to produce concrete made entirely from both CCCA and CCFA, achieving a compressive strength of 25 MPa at a dry density of 1975 kg/m³, making it suitable for lightweight structural applications.
6. The splitting tensile and flexural strengths were more significantly affected by the CCCA than the CCFA. Around 10% reduction or less in both strengths was induced by including 25% CCCA, while this reduction was caused in CCFA mixtures when 50% replacement was used. In addition, at 100% replacement level, the reduction in splitting tensile and flexural strength reached up to 27% and 41.3%, respectively, in CCCA mixtures and reached up to 18.3% and 31.1%, respectively, in CCFA mixtures.
7. The inclusion of either CCCA or CCFA negatively impacted the accuracy of the equations proposed by ACI 318 for predicting splitting tensile strength and flexural strength. This highlights the need for further research to adapt these equations, accounting for the effects of special types of aggregates, rather than relying solely on compressive strength.
8. The impact energy absorption capacity of both cylindrical and beam specimens decreased as the replacement level of CCCA or CCFA increased. At each replacement level, the reduction was less pronounced for CCFA compared to CCCA. However, for both types of aggregates, the impact energy absorption capacity declined significantly once the replacement level exceeded 25%.
9. In contrast to the other mechanical properties, CCFA demonstrated lower performance than CCCA in terms of abrasion resistance. The abrasion resistance remained within acceptable limits for concrete mixes containing up to 50% CCCA (M2 and M3) and for the mix with 25% CCFA (M6). However, higher replacement levels led to significant reductions in abrasion resistance, restricting the use of such concrete to applications exposed to abrasive forces.
10. The UPV readings decreased as the replacement levels of CCCA and CCFA increased, showing a similar reduction up to 75% for both aggregates. At the 100% replacement level, the inclusion of CCCA had a more pronounced effect than CCFA in reducing the UPV. While the UPV provided reliable trends regarding changes in concrete properties, the correlation was relatively weak, which may limit its effectiveness for precise estimation.
11. Similar to the UPV, the Schmidt rebound hammer showed lower readings as the content of CCCA or CCFA increased, with lower values observed for CCCA compared to CCFA at the same replacement level. However, unlike the UPV test, the Schmidt hammer test proved to be more reliable, offering a higher correlation with the measured properties.
12. Statistical analysis revealed that both CCCA and CCFA, along with their interaction, significantly influenced most concrete properties. However, their use led to results with greater variability compared to natural aggregates, primarily due to the non-uniform nature of the recycled materials. The greatest variability was observed in the splitting tensile strength, abrasion resistance, and impact energy tests.
13. The use of CCCA can help reduce the embodied energy of concrete compared to natural coarse aggregates, offering a more sustainable alternative. However, the inclusion of CCFA tends to increase the embodied energy due to the processing requirements. Despite this, CCFA remains a viable and sustainable alternative for natural sand, contributing to the conservation of natural resources and promoting more eco-friendly construction practices.

Finally, based on the outcomes of this study, CCCA and/or CCFA can be effectively utilized to develop low-density concrete suitable for various structural applications. Optimal performance was observed atreplacement levels of 25% for CCCA and up to 50% for CCFA, with only minimal reductions in strength. Moreover, the use of CCCA and CCFA as partial replacements for natural aggregates contributes to environmental sustainability by reducing the demand for natural resources and minimizing construction waste. However, concrete incorporating CCCA and/or CCFA may have limited suitability for applications exposed to severe abrasion or impact loads. Further research is recommended to validate the material and structural performance of CCCA and CCFA, supporting their broader adoption in the construction industry and inclusion in international design guidelines.

## Data Availability

All data generated or analyzed during this study are included in this published article.
